# Allergenic Pollen Dynamics in Continental-Climate Urban Ecosystem: Multivariate Statistical Modeling of a 24-Month Observational Study

**DOI:** 10.3390/plants15162476

**Published:** 2026-08-15

**Authors:** Gül Esma Akdoğan Karadağ

**Affiliations:** Department of Molecular Biology and Genetics, Faculty of Science and Letters, Kafkas University, Kars 36100, Türkiye; gulesmaakdogan@kafkas.edu.tr

**Keywords:** airborne pollen, pollen calendar, allergenic pollen, volumetric trap, continental climate

## Abstract

This study is the first to assess airborne allergenic pollen in Erzincan, a continental climate region with mixed native vegetation, agricultural land, and urban areas, using volumetric sampling and comprehensive statistical models. Weekly samples from 2022 to 2023 were converted to 24 h slides and examined microscopically, and concentrations were calculated as pollen/m^3^ according to REA methodology. A total of 730 days of observations were evaluated. The annual pollen integrals were calculated separately for each year, and their combined total was 22,225 pollen*day/m^3^ (2022: 8667; 2023: 13,558). Woody pollen comprised 53.67% (2022) and 51.86% (2023); herbaceous pollen comprised 46.01% (2022) and 48.07% (2023). Thirty six pollen taxa were identified; the peak months were May 2022 (24.5%) and June 2023 (27.5%). Daily total pollen concentration was correlated positively with temperature (*r_s_* = 0.33, *p* < 0.001) and weakly with wind speed (*r_s_* = 0.17, *p* < 0.001) but negatively correlated with humidity (*r_s_* = −0.26, *p* < 0.001). Precipitation showed no significant association with pollen levels (*r_s_* = −0.02, *p* = 0.13), indicating that warmer and drier conditions favor higher pollen concentrations. The LMM showed that pollen density was affected by meteorological variables as well as interannual climatic variation, with temperature being the positive predictor (*β* = 0.348; *p* < 0.001). Canonical correlation analysis revealed a significant, multidimensional relationship between meteorological variables (temperature, humidity, rainfall, wind) and the daily densities of eight pollen taxa (Wilks’ λ = 0.603; *p* < 0.001). Canonical Correspondence Analysis indicated that meteorological variables influenced pollen taxon composition (F = 20.235, *p* = 0.001), explaining 11.51% of total inertia and suggesting that additional ecological factors may also contribute. In conclusion, pollen dynamics are sensitive not only to seasonal and meteorological variables but also to interannual climate fluctuations; in particular, hot, dry periods prolong the persistence of airborne pollen, while short-term rainfall can trigger sudden pollen releases in some species.

## 1. Introduction

Pollen in the atmosphere substantially influences air quality, human health, and ecosystems [1]. A significant number of allergic diseases are triggered by airborne pollen, which is attributable to seasonal pollination [2]. Pollen grains are released into the atmosphere in large quantities by flowers as part of the plant’s natural growth cycle. This abundance of pollen in the atmosphere plays a crucial role in the processes of seed formation, crop yield, and the reproduction of wild vegetation in the biosphere [3]. The degree of allergenicity of pollen varies according to the species. Wind-pollinated plants are the primary cause of pollen allergies in individuals who are sensitive to pollen, and symptoms may include allergic rhinitis (hay fever), allergic conjunctivitis, and allergic asthma [4]. Numerous aeropalynological studies have been conducted worldwide and in Türkiye to investigate airborne pollen, particularly allergenic taxa. These studies have characterized the seasonal patterns of airborne pollen, assessed the potential impacts of allergenic pollen on sensitized individuals, and contributed to the development of pollen calendars for many regions, providing valuable information for allergy prevention and management [5,6,7,8]. In addition to describing airborne pollen seasons and developing pollen calendars, numerous studies have investigated the relationships between airborne pollen concentrations and meteorological variables. These studies have shown that meteorological factors influence the seasonal dynamics and atmospheric concentrations of airborne pollen [9,10]. The relationship between airborne pollen concentrations and meteorological variables is complex and depends on multiple interacting environmental factors. The influence of individual meteorological parameters may vary among pollen taxa and is further modified by local climatic conditions, vegetation composition, and interannual variability. Consequently, similar meteorological conditions may result in different airborne pollen responses across regions and years [8,10,11]. In addition, broader environmental drivers, including air pollution and global environmental change, may alter pollen production, seasonality, and the transport and persistence of airborne pollen [12]. Therefore, long-term aeropalynological monitoring combined with comprehensive statistical analyses across different climatic regions is essential to improve our understanding of the factors governing airborne pollen dynamics and to enhance the prediction of pollen-related exposure.

Globally, continental climate zones with large landmasses such as the Central Asian steppes, the interior of North America, and the transition zones between Eastern and Western Asia are characterized by significant annual temperature variations, low humidity, prevailing wind patterns, and irregular rainfall. These climatic conditions directly affect pollen production, release, and atmospheric transport, significantly shaping the duration, intensity, and temporal distribution of pollen seasons. Studies show that pollen seasons in these regions are longer and reach higher concentrations during specific periods [13,14]. Furthermore, in these climate zones a significant portion of the population is concentrated in urban centers, making allergen exposure even more critical from a public health perspective [15,16,17,18]. In Türkiye, Erzincan stands out as one of the centers exhibiting continental climate characteristics and has rich plant diversity and high endemism potential due to its location at the intersection of the Eastern Black Sea, Eastern Anatolia, and Central Anatolia regions. The province’s climatic characteristics, vegetation diversity, and the potential exposure of the population living in the city center to allergens make Erzincan a model area for generalizing findings to population groups living in continental climate zones [19].

This study investigates the relationships between airborne pollen and meteorological factors in Erzincan Province, an important biogeographical transition zone in Türkiye characterized by a continental climate, and presents the first volumetric aeropalynological assessment conducted in the province. In addition, by integrating complementary statistical approaches, the study provides a comprehensive evaluation of pollen–meteorology relationships. This analytical framework enables a more detailed characterization of seasonal pollen dynamics, the meteorological factors influencing them, and interannual variability, while providing a practical approach for the comprehensive statistical analysis of aeropalynological datasets from regions with similar continental climatic conditions. As part of the study, a regional pollen calendar was also developed to describe the seasonal occurrence of the major airborne pollen taxa. Consequently, the study not only provides baseline aeropalynological data for Erzincan but also contributes to a better understanding of airborne pollen dynamics in continental climate regions and offers a valuable reference for comparative aeropalynological studies conducted in similar environments worldwide.

## 2. Results

**Figure 1:**
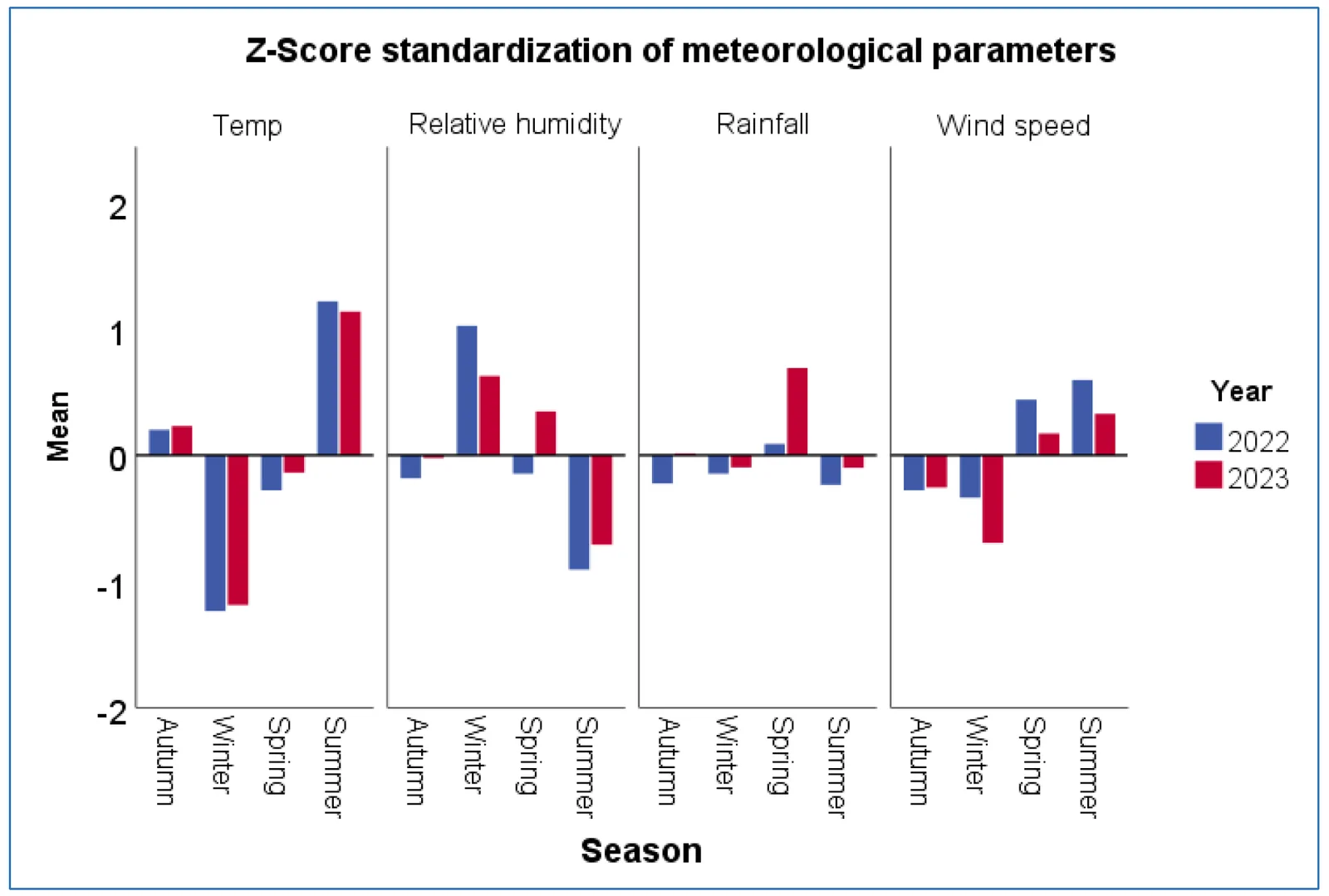
Seasonal meteorological variations during 2022–2023.

A total of 730 days of observations were evaluated during the study period (2022–2023). Daily pollen concentrations ranged from 0 to 335 pollen/m^3^, with a mean of 3.16 ± 13.43 (pollen/m^3^). Meteorological variables showed that daily mean temperature ranged from −11.1 °C to 30.5 °C, with an overall mean of 12.41 ± 9.72 °C. Relative humidity ranged from 16.1% to 94.0%, with a mean of 55.18 ± 16.69%. Daily total rainfall ranged from 0 to 36.6 mm, with a mean of 1.11 ± 3.19 mm. Average wind speed ranged from 0.1 to 4.1 m/s, with a mean of 1.05 ± 0.55 m/s. These findings reflect a typical continental climate pattern characterized by many days of low rainfall interspersed with short periods of intense rainfall (Figure 1).

**Figure 2:**
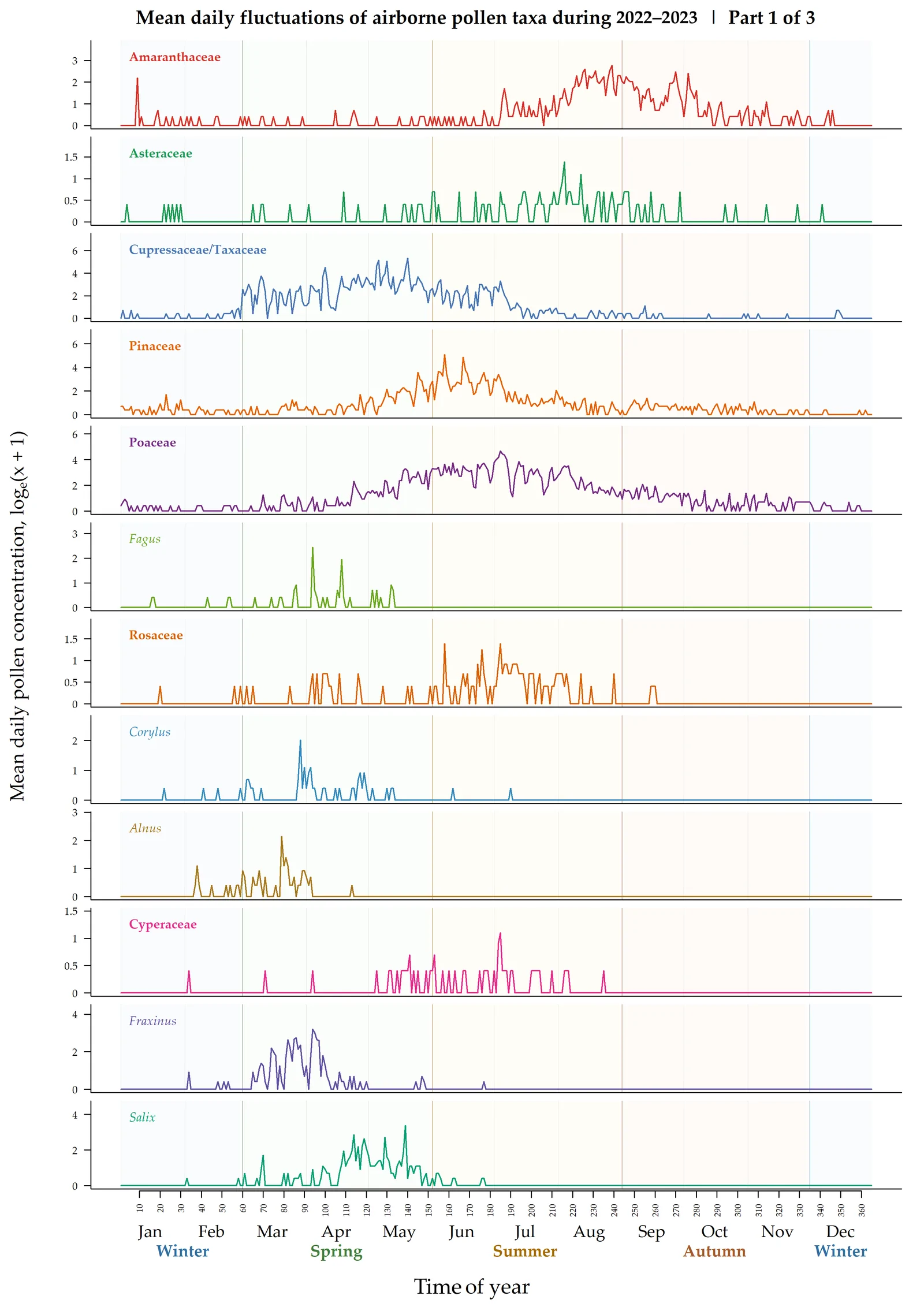

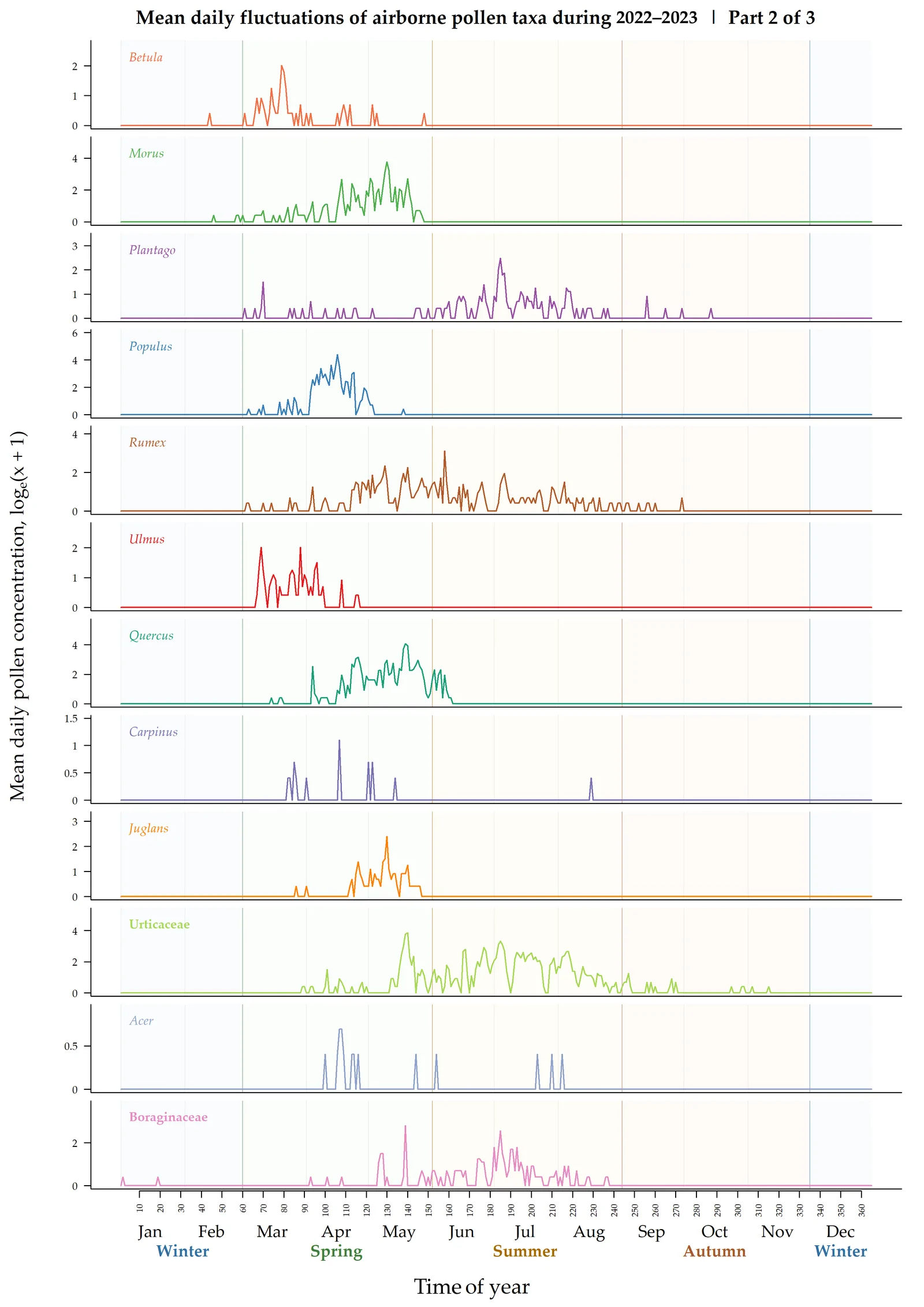

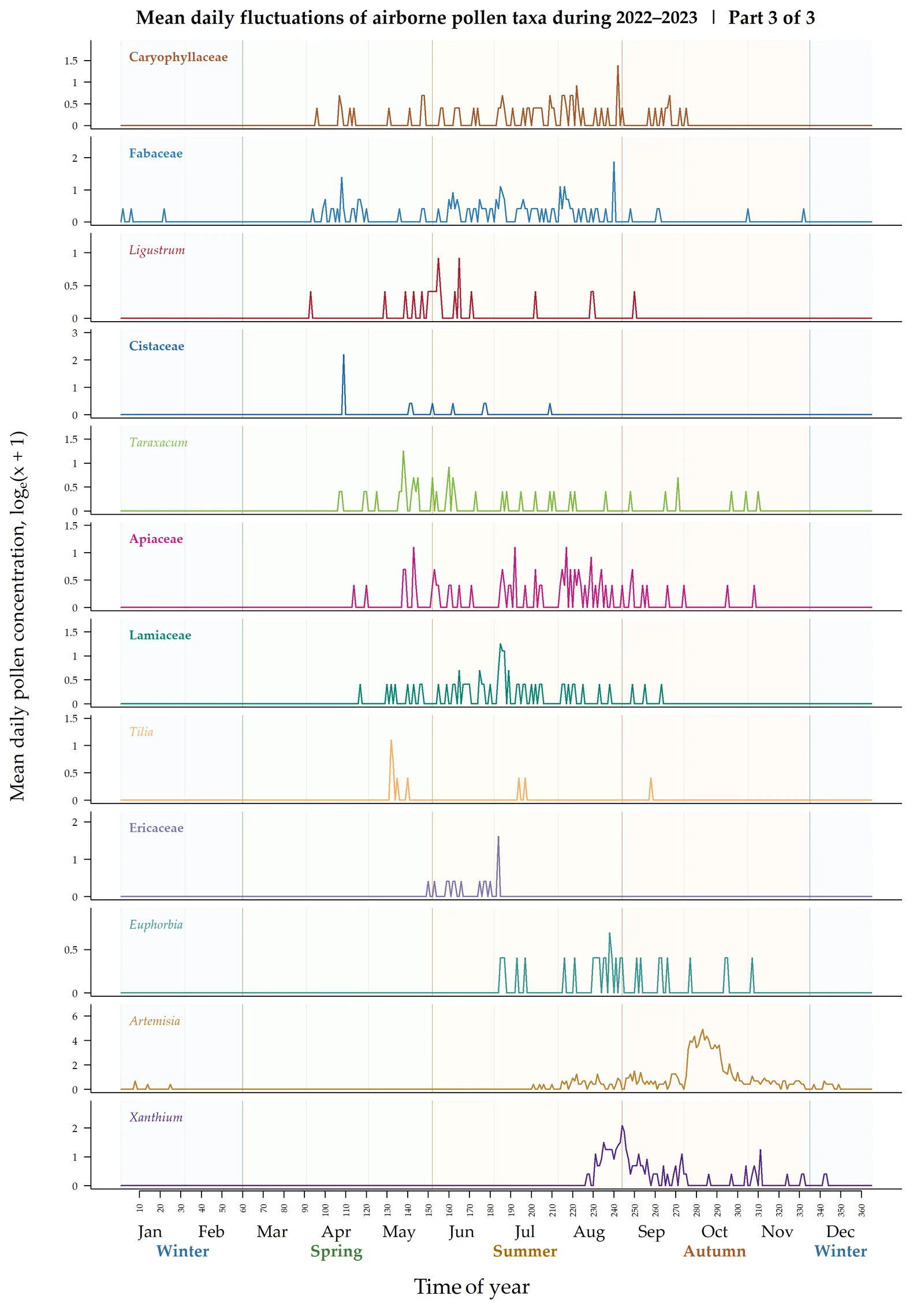
Mean daily fluctuations of airborne pollen taxa during 2022–2023.

The annual pollen integral (APIn) was 8667 pollen*day/m^3^ in 2022 and 13,558 pollen*day/m^3^ in 2023. In 2022, 53.67% of the identified pollen grains belonged to woody plants and 46.01% to herbaceous plants, whereas in 2023, 51.86% were woody and 48.07% were herbaceous. In both years, pollen from 36 taxa was identified. The month with the highest pollen concentration in Erzincan was May in 2022 (24.5%), while in 2023 it was June (27.5%). The increased number of Poaceae pollen recorded in June 2023 compared to the previous year made June the peak period for pollen concentration. In both years, woody pollen reached its maximum concentration in May, while herbaceous pollen peaked in July (Figure 2, Supplementary Materials Tables S1 and S2).

**Figure 3:**
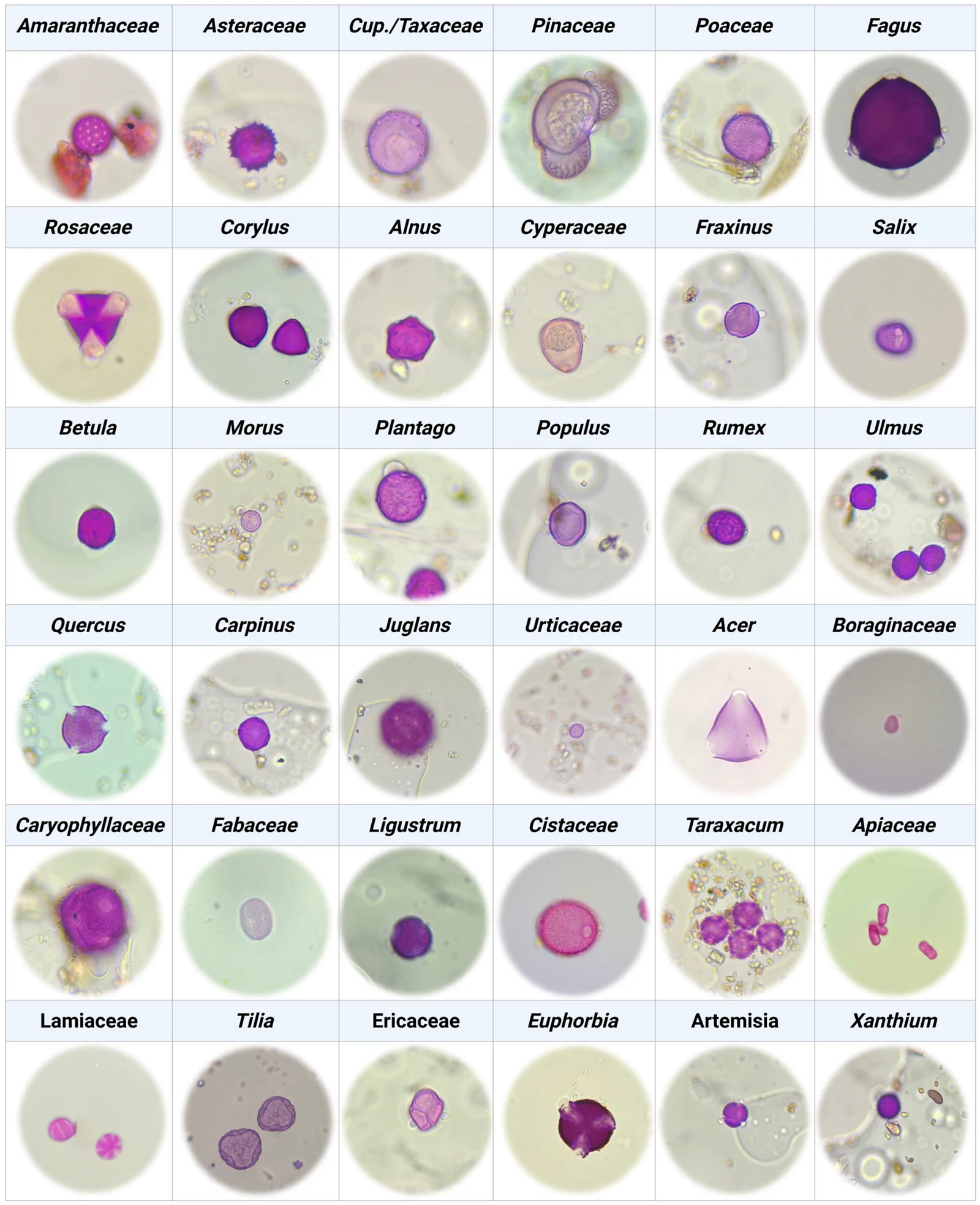
Representative microscopic images of pollen taxa identified in the atmosphere of Erzincan at 400× magnification.

Mean daily fluctuations of airborne pollen taxa in Erzincan Province revealed that the earliest pollen-producing taxa were Amaranthaceae, Asteraceae, Pinaceae, Poaceae, and Cupressaceae/Taxaceae, while the latest pollen producer was *Xanthium*. Overall, March–May showed the highest pollen concentrations for woody plants, whereas June–August were the peak months for herbaceous plants. Similarly, the seasonal dynamics of the dominant pollen taxa showed that Cupressaceae/Taxaceae attained their highest concentrations in spring, while Poaceae reached peak levels during summer period (Figure 2). Figure 2 also illustrates the atmospheric presence and relative concentrations of each taxon throughout the year. Microscopic images of the pollen taxa shown in the daily fluctuation figure and identified in the atmosphere at 40× magnification are presented in Figure 3.

### 2.1. Dominant Pollen Taxa

For Erzincan Province, dominant pollen taxa were defined as those with a concentration exceeding 3% [20]. These taxa were identified as Cupressaceae/Taxaceae (22.92%), Poaceae (21.58%), Pinaceae (11.65%), *Artemisia* (8.51%), Urticaceae (5.92%), *Quercus* (4.66%), Amaranthaceae (4.03%), and *Populus* (3.82%) (Table 1).

**Figure 4:**
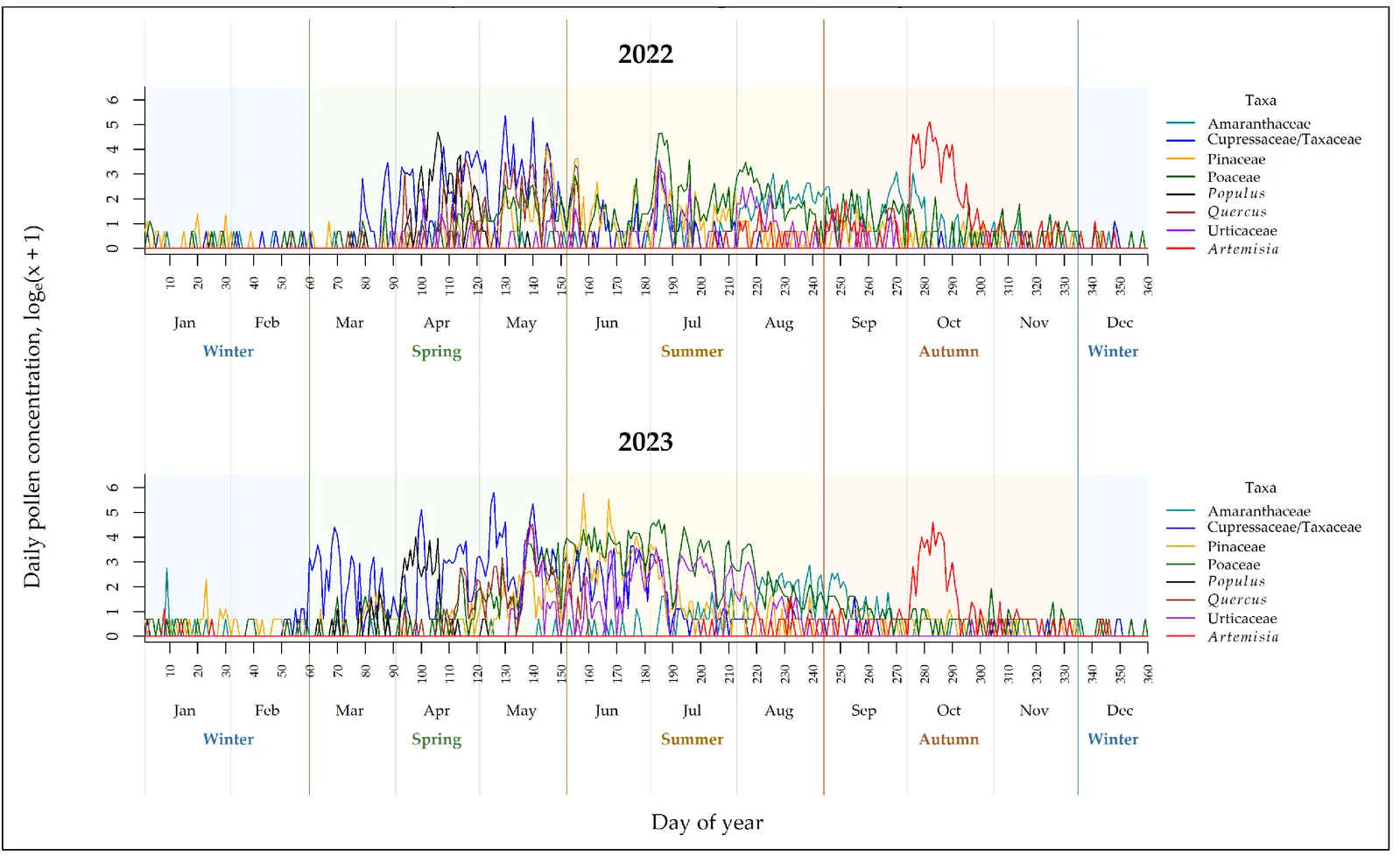
Daily fluctuations of dominant pollen taxa in 2022 and 2023.

Cupressaceae/Taxaceae was the dominant pollen taxon in the atmosphere of Erzincan. The main pollen season occurred between early March and early July, with the highest airborne concentrations recorded in May. Compared with 2022, the 2023 pollen season was longer and reached a higher peak daily concentration (Figure 4, Table 2, Supplementary Materials Figure S1).

Poaceae was the second most abundant pollen taxon in the atmosphere of Erzincan. Compared with 2022, the 2023 pollen season was characterized by a higher annual pollen integral (APIn) and higher peak daily pollen concentrations (Figure 4, Table 2, Supplementary Materials Figure S2).

Pinaceae was the third most abundant pollen taxon in the atmosphere of Erzincan. Despite the shorter duration of the main pollen season, the 2023 pollen season was characterized by a higher annual pollen integral (APIn) and higher peak daily pollen concentrations than 2022 (Figure 4, Table 2, Supplementary Materials Figure S1).

*Artemisia* was the fourth most abundant pollen taxon in the atmosphere of Erzincan. Although the main pollen season was longer in 2023, the annual pollen integral (APIn) was slightly lower than in 2022 (Figure 4, Table 2, Supplementary Materials Figure S2).

Urticaceae was the fifth most abundant pollen taxon in the atmosphere of Erzincan. Compared with 2022, the 2023 pollen season showed a substantially higher annual pollen integral (APIn), despite having a shorter main pollen season (Figure 4, Table 2, Supplementary Materials Figure S2).

*Quercus* was the sixth most abundant pollen taxon in the atmosphere of Erzincan. Compared with 2022, the 2023 pollen season had a slightly higher annual pollen integral (APIn), despite a considerably shorter main pollen season (Figure 4, Table 2, Supplementary Materials Figure S1).

Amaranthaceae was the seventh most abundant pollen taxon in the atmosphere of Erzincan. Although the duration of the main pollen season was similar in both years, the APIn was higher in 2022 than in 2023 (Figure 4, Table 2, Supplementary Materials Figure S2).

*Populus* was the eighth most abundant pollen taxon in the atmosphere of Erzincan during both study years. Although the main pollen season was longer in 2023, the annual pollen integral (APIn) was higher in 2022 (Figure 4, Table 2, Supplementary Materials Figure S1).

### 2.2. Statistical Models: Pollen Dynamics and Seasonal and Meteorological Interaction

#### 2.2.1. Spearman Correlation Analysis

The study found a significant relationship between daily total pollen concentration and meteorological variables. Pollen levels increased with rising temperatures, indicating a positive correlation between the two variables (*r_s_* = 0.33 *p* < 0.001). However, pollen levels decreased with increasing humidity, showing a significant negative correlation (*r_s_* = −0.26 *p* < 0.001). An increase in wind speed showed a weak correlation with a slight increase in pollen concentration (*r_s_* = 0.17 *p* < 0.001). Furthermore, no significant correlation was found between total rainfall and pollen density (*r_s_* = −0.02 *p* = 0.13). Furthermore, the interannual comparison clearly indicated that the rainfall (total annual precipitation) increased substantially from 253.2 mm in 2022 to 553.4 mm in 2023. A similar increase was also observed during the spring period (127.6 mm vs. 304.2 mm), coinciding with a higher total pollen load in 2023. However, subsequent monthly and daily analyses indicated that the relationship between precipitation and pollen concentrations was not consistent throughout the season, suggesting that rainfall interacted with other meteorological factors and taxon-specific phenological responses rather than acting as an isolated driver of pollen variability. Additionally, significant correlations in the expected directions were observed among meteorological variables (*p* < 0.001); notably, temperature and humidity showed a negative correlation (*r_s_* = −0.731 *p* < 0.001). These findings suggest that rising temperatures may increase pollen density, whereas rising humidity may reduce it. Wind speed appears to have a supportive, but limited, effect on pollen transport.

**Figure 5:**
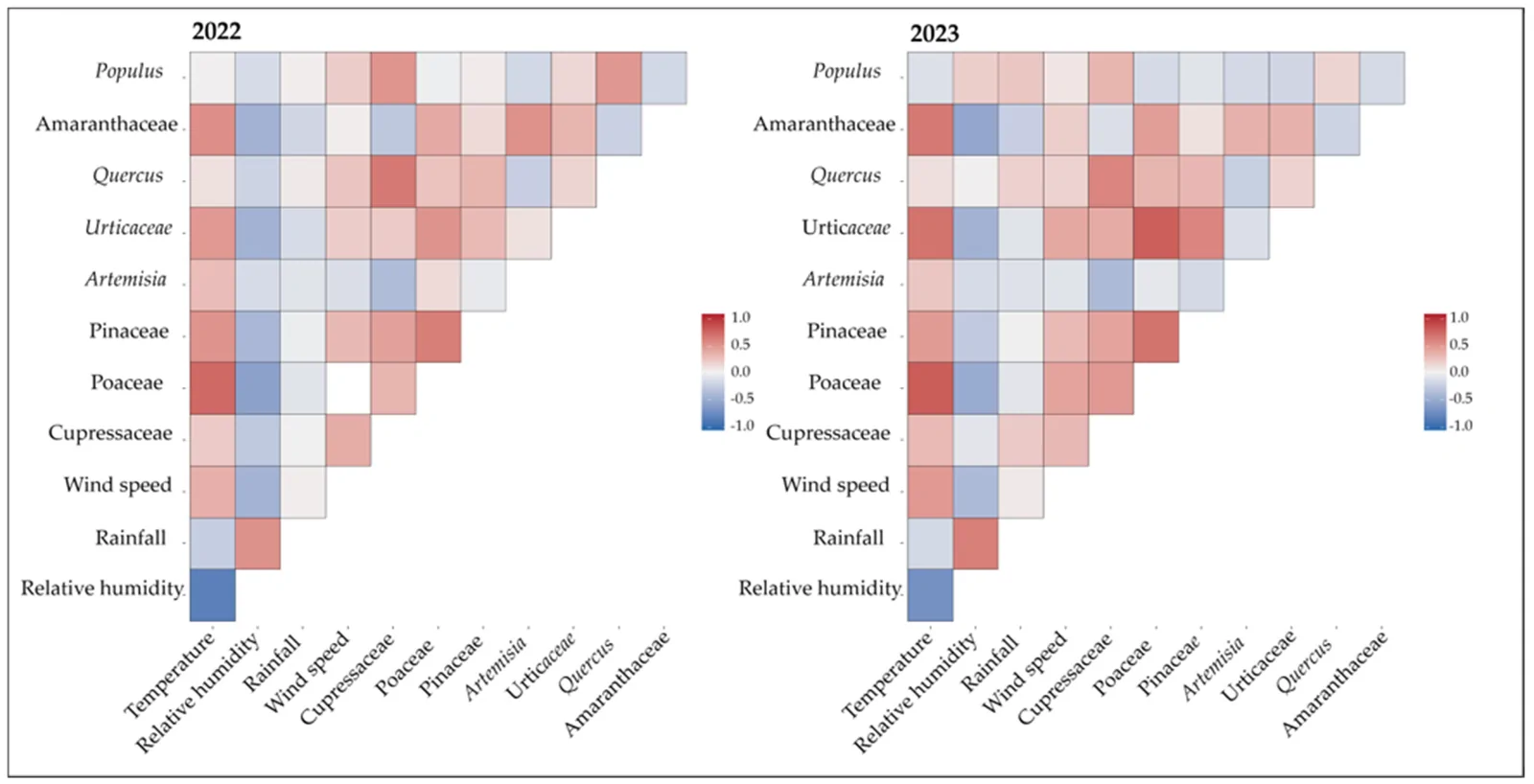
Correlation matrix of daily pollen concentrations and mean daily meteorological variables (2022–2023).

The relationships between daily average concentrations of the eight dominant pollen taxa in the Erzincan atmosphere (Cupressaceae/Taxaceae, Poaceae, Pinaceae, *Artemisia*, Urticaceae, *Quercus*, Amaranthaceae, and *Populus*) and meteorological data for 2022 and 2023 were evaluated using Spearman rank correlation. Overall, temperature showed positive correlations with pollen concentrations while relative humidity exhibited negative correlations, indicating higher pollen levels under warmer, drier conditions. Precipitation generally had weak or negative effects and wind speed effects were variable; although the main patterns were consistent between 2022 and 2023, correlation magnitudes differed between years. Significant positive correlations (*p* < 0.001) were observed between the annual mean concentrations of the dominant pollen taxa and temperature; however, *Populus* exhibited a significant negative correlation with temperature in 2023 (*p* < 0.05), whereas *Quercus* showed a weak, non-significant association. Although a significant negative correlation with relative humidity was observed (*p* < 0.01), the correlations for Pinaceae, Poaceae, Amaranthaceae, and Urticaceae were particularly pronounced. Daily rainfall was significantly positively correlated with the mean pollen concentrations of Cupressaceae/Taxaceae, *Populus*, and *Quercus*, whereas Amaranthaceae, *Artemisia* and Urticaceae exhibited significant negative correlations (*p* < 0.01). Dominant pollen taxa showed a significant positive correlation with wind speed (*p* < 0.01), whereas *Artemisia* exhibited a significant negative correlation in 2022 (*p* < 0.01, Table 3, Figure 5).

#### 2.2.2. Multiple Linear Regression (MLR) and General Linear Model (GLM) Analysis

The multiple linear regression model established to explain the pollen concentration is statistically significant (F (4, 5835) = 147.99; *p* < 0.001) and the model variance is R^2^ = 0.092 (adj. R^2^:0.091). In the general linear model (GLM), pollen concentration was modeled with species and meteorological variables as fixed effects, with species included to account for interspecific differences. The effects of meteorological variables on pollen density, species differences, and species × meteorological interactions were assessed. Statistical modeling indicated that pollen density was significantly influenced by both meteorological variables and interspecific differences (F(39, 5839) = 10.85, R^2^ = 0.068, adj. R^2^ = 0.062, η^2^_p_ = 0.068, *p* < 0.001). Given that MLR and GLM do not fully account for the repeated structure of the dataset or the temporal autocorrelation of daily pollen observations, and explain only a small proportion of the variance, their outputs are presented as supplementary supporting analyses rather than as the primary basis for inference (Supplementary Materials S3).

#### 2.2.3. Linear Mixed Model (LMM) Analysis

Candidate models with different fixed-effect structures were compared to determine the best-fitting model for log pollen number using AIC, BIC, marginal R^2^ and AR(1) rho values (Table 4). All models included an AR(1) covariance structure to account for temporal autocorrelation between repeated observations. Taxa were also evaluated as a random intercept; however, the estimated random-intercept variance was effectively zero, and conditional R^2^ did not differ from marginal R^2^. Therefore, taxon was retained as a fixed effect in the final model. The model including year, season, taxon, active pollen release period and meteorological variables showed the best fit, with the lowest information criteria and the highest marginal R^2^. Therefore, this model was selected as the final model. The marginal R^2^ indicated that fixed effects explained 35.8% of the variance in log pollen number; however, this explanatory power reflected season/taxon identity rather than meteorological predictors, since the active pollen release period (Aprp) is essentially an intraseasonal indicator. The AR(1) rho value indicated positive autocorrelation between consecutive observations (*p* < 0.001).

The linear mixed model showed that pollen density was significantly affected by both meteorological variables and interannual climatic differences. Among the fixed effects, temperature had the strongest positive effect on pollen density (*β* = 0.348; *p* < 0.001). Rainfall also had a positive and significant effect on pollen density (*β* = 0.031, *p* = 0.042), while humidity and wind were not significant (*p* > 0.05). When the year effect was included, average pollen levels in 2022 were significantly lower than in 2023 (*β*= −0.124, *p* < 0.001). However, year–meteorological interactions show that the effect of meteorological variables varied from year to year: in 2022, the effect of temperature on pollen was significantly weaker compared to 2023 (*β* = −0.220; *p* < 0.001), while the effect of humidity was more negative (*β* = −0.164; *p* < 0.001). In 2022, precipitation showed a positive trend with pollen density (*β* = 0.588, *p* = 0.054). Wind, however, had no significant effect that year (*β* = −0.009, *p* = 0.688). The LMM analysis revealed that the seasonal responses of dominant pollen taxa differed significantly across specific seasonal periods (Table 5). Significant interactions were detected for *Artemisia*–autumn, Cupressaceae/Taxaceae–spring, and Poaceae/*Quercus*–summer (*p* < 0.05), indicating taxon-specific seasonal variation in atmospheric pollen concentrations. The non-significant year effect between 2022 and 2023 suggests that these seasonal patterns were not attributable to interannual variability, but rather reflected responses predominantly driven by seasonal dynamics. In this context, alongside the significant positive associations observed for *Artemisia*, Cupressaceae/Taxaceae, Poaceae, and Pinaceae, the significant negative interaction between *Quercus* and summer (*β* = −0.55, *p* = 0.028) supports a phenological structure in which *Quercus* pollen concentrations peak in late spring and subsequently decline during the summer period.

Additional sensitivity analyses were conducted to evaluate whether the meteorological effects were influenced by temporal covariance. The day index was used to define the repeated-measures structure within the AR(1) covariance model rather than as an ordinary fixed co-factor. Alternative models excluding season and excluding both year and season indicated that temporal structure contributed to model fit, but did not solely determine the pollen–meteorology relationships. VIF values showed no problematic multicollinearity among meteorological predictors when evaluated separately (temperature = 2.18, humidity = 2.64, rainfall = 1.27, wind speed = 1.16), although temperature showed moderate inflation in the full model (VIF = 5.49). Complementary CCA and CCorA analyses indicated that the meteorological signal partly overlapped with seasonal phenology, but remained detectable after adjustment for year, season and day.

When seasonal effects are considered, pollen–climate relationships do not remain constant throughout the year but are shaped by seasonal dynamics. Accordingly, the differing meteorological effects in 2022 and 2023 were evaluated across three main seasons.

1. *Early season (early spring; March–April)*

Increased temperatures during the early season create a critical threshold at which plants phenologically transition to pollen production. The effect of warming on pollen density in 2023 was substantial, indicating that an early-warming spring accelerated pollen release. Although the temperature effect was significant in 2022, it was weaker, suggesting that spring 2022 was cooler and that pollen production started later.

2. *Peak season (late spring–early summer; May–June)*

During the peak season, temperatures are generally high and humidity is low. Although rising temperatures produced a significant increase in pollen release in 2023, humidity had no statistically significant effect that year; in 2022, however, humidity had a stronger, significantly negative effect on pollen density. This pattern suggests that peak season 2022 was relatively more humid, and higher humidity shortened pollen suspension time in the atmosphere, reducing measured pollen densities.

3. *Late season (late summer–autumn; August–October)*

While pollen production generally declines during the late season, some species such as Amaranthaceae and *Artemisia* remain actively pollen-productive. The impact of rainfall on pollen density was greater in 2022 than in 2023. This suggests that late-season rainfall can trigger pollen production in certain species, and that temperature increases following short rain events particularly amplify late-season pollen release.

#### 2.2.4. Canonical Analyses of Pollen–Meteorology Relationships

Canonical Correlation Analysis (CCorA) revealed a significant multidimensional linear relationship between meteorological variables—temperature, humidity, precipitation and wind—and the daily densities of eight pollen taxa (Wilks’ λ = 0.603, F = 12.175, *p* < 0.001). The first canonical function represented the dominant association between the two variable sets (eigenvalue = 0.560, canonical correlation = 0.599, r^2^ = 0.359), accounting for 89.99% of the canonical relationship. This function explained 42.39% of the variance in meteorological variables and 16.10% of the variance in pollen taxa. The first canonical function was mainly characterized by a temperature–humidity gradient, indicating that higher temperature and lower humidity were associated with variation in daily pollen taxon densities.

**Figure 6:**
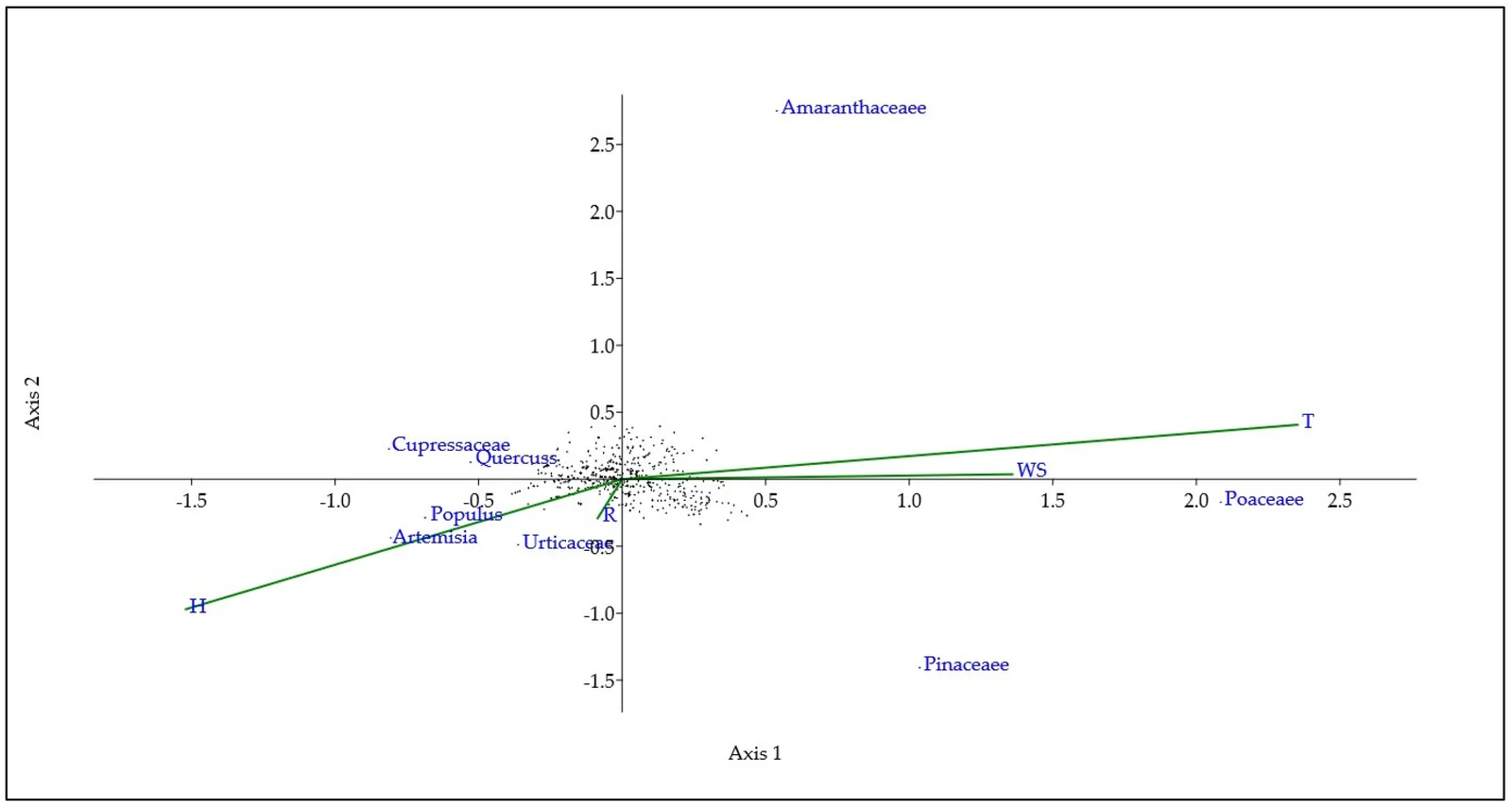
CCA ordination diagram showing the relationships among pollen taxa, samples, and meteorological variables. Black points represent daily samples, blue labels indicate pollen taxa, and green vectors represent meteorological variables. The direction of the vectors indicates the increasing gradient of each meteorological variable, while the relative positions of pollen taxa and samples reflect their associations with the first two CCA axes. CCA, Canonical Correspondence Analysis; T, temperature; H, humidity; R, rainfall; Ws, wind speed.

To further examine the pollen–meteorology relationship within an ecological ordination framework, Canonical Correspondence Analysis (CCA) was subsequently performed. The overall CCA model was statistically significant according to the permutation test (F = 20.235, *p* = 0.00137), indicating that meteorological variables contributed to the variation observed in pollen taxon composition. The constrained component accounted for 11.51% of the total inertia, whereas 88.49% of the variation remained unconstrained, suggesting that additional unmeasured environmental or ecological factors may also be involved in pollen distribution patterns. The first canonical axis represented the main constrained gradient, explaining 74.26% of the species–environment relationship and 8.55% of the total species variation. This axis was mainly associated with temperature in one direction and humidity/rainfall in the opposite direction. The second axis explained 19.23% of the species–environment relationship and was primarily related to wind. The first three canonical axes were statistically significant, whereas four axis was not significant. All VIF values were below 3, suggesting that there was no evidence of serious multicollinearity among the climatic predictors. These CCA results were consistent with the CCorA findings and provided a complementary ordination-based interpretation of the meteorological gradients associated with pollen taxon composition (Figure 6, Table 6).

In conclusion, pollen seasons in high-altitude continental climates are sensitive to both meteorological factors and interannual climate variability. Hot, dry periods prolong airborne pollen and increase allergen loads, while brief rainfall events can trigger sudden pollen releases in some species. Therefore, allergy-management strategies and early-warning systems should account for sharp seasonal transitions, year-to-year climate variability, and species-specific ecological responses in continental climates.

## 3. Discussion

The determination of airborne particles is important for identifying particles that cause respiratory diseases. Due to the differences in climate and vegetation characteristics across regions, it is necessary to conduct atmospheric studies at the level of regions, provinces, cities, and districts. The airborne pollen in Erzincan, located in eastern Türkiye and situated in a significant transition zone between different geographical regions of the country, where a continental climate predominates, was investigated in this study using the volumetric method. A two-year average APIn of 11,113 pollen*day/m^3^ across 36 taxa was recorded in Erzincan, with 52.57% of the detected pollen belonging to woody taxa. In other studies, conducted in areas where a continental climate predominates in Eastern Anatolia, this proportion varied according to the flora characteristics of the provinces. Woody taxa accounted for 58.20% in Van, 62.66% in Mardin, and 76.39% in Elazığ, similarly to those in Erzincan, whereas lower proportions were observed in Kars (29.31%) and the Sarıkamış district of Kars (36.34%). The lower values in Kars and Sarıkamış are attributed to the steppe-dominated flora of these areas. In other studies, conducted across Türkiye, pollen from woody taxa was generally found in higher proportions, consistent with the findings in Erzincan [20,21,22,23,24,25,26].

The results of this study show that woody plant taxa are more abundant during the spring period in Erzincan, whereas herbaceous taxa dominate in the summer months (Supplementary Materials Tables S1 and S2). This pattern is expected, as anemophilous trees begin their vegetation period earlier, while annual plants release their pollen in middle to late summer depending on seed development and life cycles. Among woody plants, Cupressaceae/Taxaceae, *Populus*, *Pinus*, *Quercus*, and *Morus* have been identified in many regions as characteristic early-flowering taxa [26,27,28,29]. The onset and peak of pollination for early-flowering woody taxa vary among climatic regions. In Mediterranean climates, pollination generally begins in January and peaks between March and April, whereas in colder continental regions it starts later [9,30,31,32]. Accordingly, the relatively later occurrence of early-flowering taxa in Erzincan is likely associated with its high-altitude continental climate. The detection of pollen from taxa outside their typical flowering periods or from predominantly entomophilous species most likely reflects low airborne pollen concentrations resulting from long-distance atmospheric transport, resuspension of previously deposited pollen by wind or human activities, and incidental wind dispersal [33,34]. In addition, pollen from the families Cupressaceae, Poaceae, Urticaceae, and Amaranthaceae exhibits long and continuous pollen seasons, which can be attributed to their high species diversity and certain limitations in pollen identification [28].

Among the dominant pollen taxa in the Erzincan atmosphere, Cupressaceae/Taxaceae family are reported as the main pollen type primarily in early spring, though they are also observed during winter [28,35,36,37,38]. The pollen grains of the *Cupressus* and *Juniperus* genera, which belong to the Cupressaceae family, have been identified as abundant allergens in the atmosphere of the Mediterranean region [7,39,40]. Studies conducted in cities with Mediterranean climates in France, Italy, Spain, and Türkiye have documented that Cupressaceae and Taxaceae pollen grains occur at high concentrations throughout the year [12,27,29,35,39,40,41]. Similarly, in Colombia, Cupressaceae and Taxaceae pollen have been observed at high concentrations [42]. *Cupressus* species are known for their high pollen production and strong allergenic potential. Studies have shown that members of the Cupressaceae family are responsible for allergic reactions such as rhinitis, asthma, and conjunctivitis in sensitive individuals [43,44,45]. Although Erzincan is characterized by a predominantly continental climate, Cupressaceae/Taxaceae pollen was observed at high concentrations throughout the year, particularly in spring and summer. Members of these families are present in Erzincan not only in natural distributions but also in landscaping, which contributes to their continuous presence in the atmosphere. Studies conducted in neighboring provinces have also identified Cupressaceae/Taxaceae among the dominant pollen taxa, similar to those in Erzincan [26,32,46,47,48]. In Erzincan, May represents a period of high allergy risk due to Cupressaceae/Taxaceae pollen, and exposure should ideally be minimized during this time.

The Poaceae family, which includes numerous wild grass species and whose pollen can be identified at the family level, was observed at high concentrations throughout the year. It was identified as the second most abundant pollen taxon in the Erzincan atmosphere. According to the Coordination of Information on the Environment (CORINE) land cover map of Erzincan, the province’s area includes natural grasslands (18.8%) and pastures (2.9%), where Poaceae members are present throughout the year. The steppe vegetation of the region is a major factor contributing to the high and year-round presence of Poaceae pollen [49]. Poaceae pollen is among the most abundant and highly allergenic pollen both in Anatolia and worldwide [20,21,22,27,40,50,51,52,53].

Pinaceae species are wind-pollinated and produce a substantial amount of pollen. Traditionally, pine pollen has been considered non-allergenic. Despite its abundance, pine pollen is regarded as non-allergenic primarily due to factors such as the large size of the pollen grains, low protein content, and the presence of a waxy hydrophobic layer that inhibits protein release [54,55]. Members of the Pinaceae family are widely distributed naturally in Türkiye and are also commonly used in afforestation and landscaping projects [56]. Examining the seasonal distribution of *Pinus* pollen across Türkiye, atmospheric pollen concentrations typically peak in May, followed by April and June [46]. In Erzincan, Pinaceae, the third most abundant pollen taxon, reached its highest concentration in June. In regions influenced by the Mediterranean climate, Pinaceae pollen peaks in March and April [21,27,28,35,53,57], whereas in cooler regions, peaks occur in May [2,20,48,58], and in higher-altitude areas with a continental climate, such as Erzincan, the peak is observed in June [26,32,47,50]. Although Pinaceae pollen is not highly allergenic, June can be considered a period when caution is recommended for this taxon.

The fourth most abundant pollen taxon in Erzincan, *Artemisia*, is a representative of the Irano-Turanian phytogeographic region and produces a large number of pollen grains [2,59]. In the Northern Hemisphere, pollen from *Artemisia* species is a significant cause of respiratory allergies. Peak *Artemisia* pollen levels are generally recorded between July and October [59]. In Erzincan, *Artemisia* pollen appears in July and reaches its highest concentration in October, making October a high-risk month for *Artemisia*-related allergies in the region. Other studies conducted in regions of Türkiye characterized by a predominantly continental climate have also observed that *Artemisia* pollen is most abundant between July and October. Specifically, peak months have been reported as July in Kars, August in Ardahan (Posof), July–August in Van, October in Mardin, August in Kastamonu, and October in Siirt [20,21,22,26,27,32].

Members of the Urticaceae family, including *Urtica* and *Parietaria* taxa, are also distributed in Erzincan [60]. These two genera cannot be distinguished under a microscope and are therefore identified at the family level [61]. *Parietaria* is the main allergenic genus within the Urticaceae family [4,62]. Urticaceae members are mainly distributed in the Mediterranean region, but their range has expanded to more northern and colder areas due to global warming [63]. *Parietaria* is known to initiate pollination in early spring, with its main pollen season extending through spring and summer in Mediterranean regions [64]. The relatively early Urticaceae peak observed on 20 May 2023 may be related to the contribution of *Parietaria* pollen. Similarly to in Erzincan, aerobiological studies conducted in Türkiye and worldwide frequently report Urticaceae among the dominant pollen taxa, posing a risk to sensitive individuals [7,21,32,35,47,64,65].

Oak (*Quercus*) belongs to the order Fagales, which also includes birch, alder, hazelnut, and hornbeam species, whose pollen grains are strong triggers of spring pollinosis in Central Europe [66]. Oaks are among the most widespread deciduous trees, occurring in Europe, North America, and parts of Asia and Africa. Türkiye is one of the richest regions in the world in terms of *Quercus* taxa, with 39 documented species [32,67]. According to the CORINE land cover map of Erzincan, broad-leaved forests containing oak trees are observed. Consequently, *Quercus* pollen ranks sixth among the dominant pollen taxa. Studies conducted in neighboring provinces have also reported *Quercus* as a dominant pollen [20,28,32,46,47].

Pollen from the Amaranthaceae family was identified as the seventh dominant taxon in Erzincan. In Türkiye, the seasonal distribution of Amaranthaceae pollen indicates that it is a dominant taxon during summer and autumn across almost all regions [56]. Among the Amaranthaceae, species of the genera *Chenopodium*, *Salsola*, and *Amaranthus* are major triggers of summer allergies in temperate and arid regions such as western USA, Australia, and the semi-desert areas of Saudi Arabia, Kuwait, India, and Iran. Desertification across large areas of the Mediterranean basin has increased the prevalence of these weeds, causing symptoms in sensitive individuals even at low pollen concentrations [68,69].

*Populus* pollen was identified as one of the dominant taxa in Erzincan. In Türkiye *P. alba* L., *P. tremula* L., *P. nigra* L., *P. euphratica*, *P. usbekistanica*, and *P. × canescens* (Ait.) naturally occur. Due to the economic value of poplar trees, many poplar hybrids have been introduced and cultivated across Türkiye [56]. Atmospheric pollen studies in Türkiye have reported *Populus* pollen as dominant, particularly in the northern and eastern regions [26,46,48]. Globally, it has been observed as dominant in areas such as Argentina, Croatia, Portugal, and Central Spain [40,53,65,70]. The period during which *Populus* pollen is present in the atmosphere generally coincides with March, April, and May, both in Erzincan and elsewhere in Türkiye and the world [26,40,46,48,53,65]. The allergenicity of *Populus* pollen is generally low [48]. In Erzincan, *Populus* pollen reached its peak in April. Therefore, April is recommended as a period of caution for sensitive individuals in Erzincan.

The definition of the MPS can substantially influence the estimated season boundaries and duration, and previous studies have shown that no single method is equally suitable for all pollen taxa and atmospheric conditions [5,71,72]. In the present study, the Andersen 2.5–97.5% method resulted in relatively long MPS durations for some taxa, particularly Pinaceae and Poaceae, likely due to the inclusion of prolonged periods of low and sporadic pollen concentrations [73]. Therefore, the 3d5 concentration-threshold approach was additionally applied as a complementary method [74]. Compared with the Andersen method, the 3d5 definition resulted in shorter MPS durations for several taxa. For example, the Poaceae MPS was reduced from 225 to 120 days in 2022, while the MPS identified for *Populus* corresponded to the main period of elevated pollen concentrations observed in the daily pollen concentration pattern. These results indicate that part of the extended MPS duration obtained with the Andersen method may be associated with prolonged periods of low and sporadic pollen occurrence.

Pollen concentration in a region is influenced not only by the local flora but also by meteorological factors that affect pollen release and dispersal [4]. It is known that water loss increases tension in the cell walls of anthers, causing them to rupture and release pollen; this mechanism is largely associated with changes in air humidity and temperature [75]. Statistical analyses identified temperature as the strongest factor increasing pollen density. In general, air temperature significantly affects pollen seasonality, producing earlier and longer pollen seasons and higher pollen concentrations. Additionally, short-term rainfall has been shown to cleanse the atmosphere of pollen, with both rainfall and humidity exerting negative effects on airborne pollen presence [75,76,77,78]. The significant positive correlation between temperature and the taxa particularly Poaceae was consistent with studies from continental-climate regions reporting a temperature pollen relationship (*p* < 0.01). This finding aligns with numerous studies showing that pollen production and release are triggered by temperature [79,80,81]. In Erzincan, temperature exhibited the expected positive effect on dominant taxa except for *Populus.* The negative correlation observed between temperature and *Populus* pollen (*p* < 0.01) can be explained by the early spring phenology of the genus. Pollen release in *Populus* species typically begins 10–20 days after reaching low temperatures of 4–5 °C, during a relatively warm period [82]. Bonini et al. [81] reported that temperature stimulated pollen production at the beginning of the main pollen season (MPS) for Urticaceae and Poaceae, but that extreme temperature increases during the MPS led to significant decreases in pollen density. This indicates that pollen density is influenced not only by conditions at seasonal onset but also by meteorological changes within the season [83]. Statistical analyses also showed a positive correlation between average annual temperature and pollen in Pinaceae, whereas during the MPS the correlation was negative (*p* < 0.01), demonstrating the importance of intraseasonal dynamics.

The effect of precipitation on pollen in the atmosphere varies. Increased rainfall has a short-term effect that can lead to lower pollen concentrations due to the “wash out” effect. The long-term effects of precipitation differ for trees and weeds [83]. The positive correlation observed in this study between precipitation and dominant early-spring woody taxa (*Populus*, *Quercus*) as well as year-round taxa with long MPS duration (Cupressaceae/Taxaceae) is consistent with Schram, who reported positive correlations between precipitation and trees or taxa with extended MPS. Despite substantial differences in meteorological conditions between 2022 and 2023, airborne pollen concentrations showed variable responses across temporal scales. Although higher annual and spring precipitation in 2023 coincided with a greater total pollen load, the decline in pollen concentrations during April despite increased precipitation indicates that rainfall alone cannot explain the observed variability. This is consistent with previous studies showing that precipitation can have both positive and negative effects on airborne pollen depending on its timing, intensity, and taxonomic context, whereas temperature generally has a more consistent influence on pollen production and season intensity [81,]. Humidity has a negative effect on airborne pollen concentration. High humidity can increase the moisture content and weight of pollen grains, lowering airborne pollen levels and thus limiting their dispersal and residence time in the atmosphere. Conversely, low humidity conditions can facilitate pollen release and transport, leading to increased airborne pollen concentrations. Li [84] reported that a 1% increase in relative humidity was associated with a 4% decrease in the risk of allergic rhinitis. Statistical analyses revealed that humidity had a negative impact on pollen concentration in the atmosphere in the studied region. Similar results were observed in studies in regions with continental climates regarding the relationship between pollen and meteorological factors [20,21,32,40,47]. Contrary to our findings, a study in Central Spain reported a positive correlation between *Populus* pollen and temperature, and a negative correlation with humidity. In the same study, the relationships between Cupressaceae, Poaceae, Pinaceae, Urticaceae, *Quercus*, and Amaranthaceae pollen and meteorological variables were similar to those observed in this research. Similarly, a study in Medellín, Colombia, found positive correlations between Urticaceae, Cupressaceae, *Pinus*, and Poaceae pollen concentrations and temperature and wind speed, and a negative correlation with humidity [42]. Although some studies have reported opposite patterns concerning temperature and humidity [21,28]. When detailed, multilevel, and multivariate statistical analyses were considered together, it was seen that the temperature–humidity axis emerged as the primary determinant of pollen composition and daily pollen densities. In continental climates, the relationship between pollen and meteorological variables varies by species and from year to year. Studies indicate that temperature is the strongest driver of increased pollen density, and that pollen release rises markedly during hot, dry periods—particularly for highly allergenic taxa such as Poaceae. While humidity was not generally a significant factor, its negative effect was more pronounced in 2022, when pollen was removed from the atmosphere more rapidly during wetter periods. Although rain generally clears pollen from the atmosphere, the observed increase in Cupressaceae/Taxaceae pollen after rainfall suggests that short rain events in continental climates can trigger pollen surge in some species. This situation reveals that variability in annual climate patterns in continental climates directly affects pollen phenology and the seasonal dynamics of airborne pollen. Higher temperatures may alter the duration of the pollen season, consequently changing the period during which individuals are exposed to airborne allergens and plant distribution. Climate change has multiple interconnected potential consequences on respiratory allergies and related diseases.

## 4. Materials and Methods

### 4.1. Sampling Area

Erzincan is located in the Upper Euphrates Basin in the northwestern part of Eastern Anatolia, between 39°02′–40°05′ N latitude and 38°16′–40°45′ E longitude. Phytogeographically, the province lies within the Irano-Turanian region; however, due to its proximity to the Euro-Siberian region and its position on the Anatolian Diagonal, which plays a key role in the distribution of plants in Anatolia, it has remarkably rich plant diversity [85]. Erzincan has a continental climate. While the vegetation cover mainly consists of short-lived herbaceous plants, tree species such as oak (*Quercus*), hornbeam (*Carpinus*), ash (*Fraxinus*), and Scots pine (*Pinus sylvestris*) are found around Refahiye and Kemah. Alpine meadows and degraded forests are also among the habitats present in the province [82,85]. Erzincan hosts about 2500 plant species, 596 of which are endemic, with 64 species being endemic exclusively to Erzincan. The flora of the province is particularly rich in genera such as *Astragalus* L., *Acantholimon* Boiss., *Allium* L., *Galium* L., *Hypericum* L., *Onobrychis* Mill., *Onopordum* L., *Potentilla* L., *Salvia* L., *Silene* L., *Tanacetum* L., *Thymus* L., and *Verbascum* L., as well as members of the Rosaceae family, especially its woody and shrubby representatives. Besides its mountainous terrain, large areas from low to high altitudes are dominated by steppe vegetation [82].

**Figure 7:**
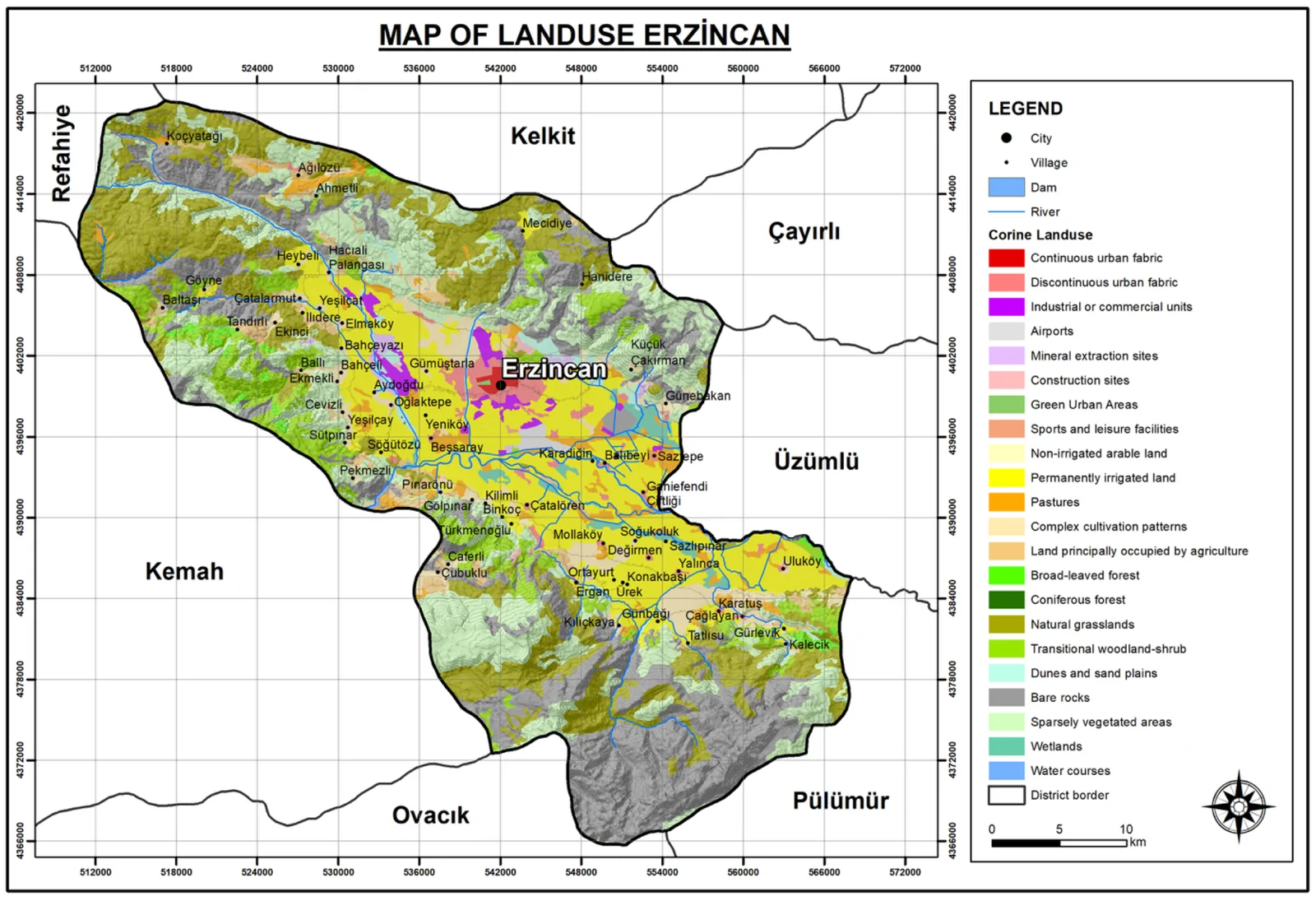
CORINE land cover map of Erzincan Province.

In addition to the floristic features of Erzincan, land cover data were used to relate the atmospheric pollen taxa with the existing vegetation. CORINE Land Cover 2018 data were applied for this purpose. According to the CORINE data for the study area, the largest land cover classes are natural grasslands (18.81%), bare rocks (20.53%), sparsely vegetated areas (19.79%), and permanently irrigated lands (17.01%). In terms of pollen production, natural grasslands and pastures (2.92%), complex cultivation patterns (4.37%), and agricultural areas associated with natural vegetation (2.45%) are important sources of wind-transported herb pollen (e.g., Poaceae, *Artemisia*). Broad-leaved forests (0.58%) and coniferous forests (0.02%) represent limited but locally influential potential sources of tree pollen. Transitional woodland-shrub areas (7.00%) may also host temporary and diverse plant species, thereby seasonally influencing the occurrence of different pollen types in the atmosphere. In contrast, urban and artificial surfaces, covering approximately 3.2% of the total area, have a relatively small contribution to pollen production. These data indicate that, in the region, herbaceous and agricultural sources are likely to dominate the atmospheric pollen composition (Figure 7).

### 4.2. Airborne Pollen Identification

**Figure 8:**
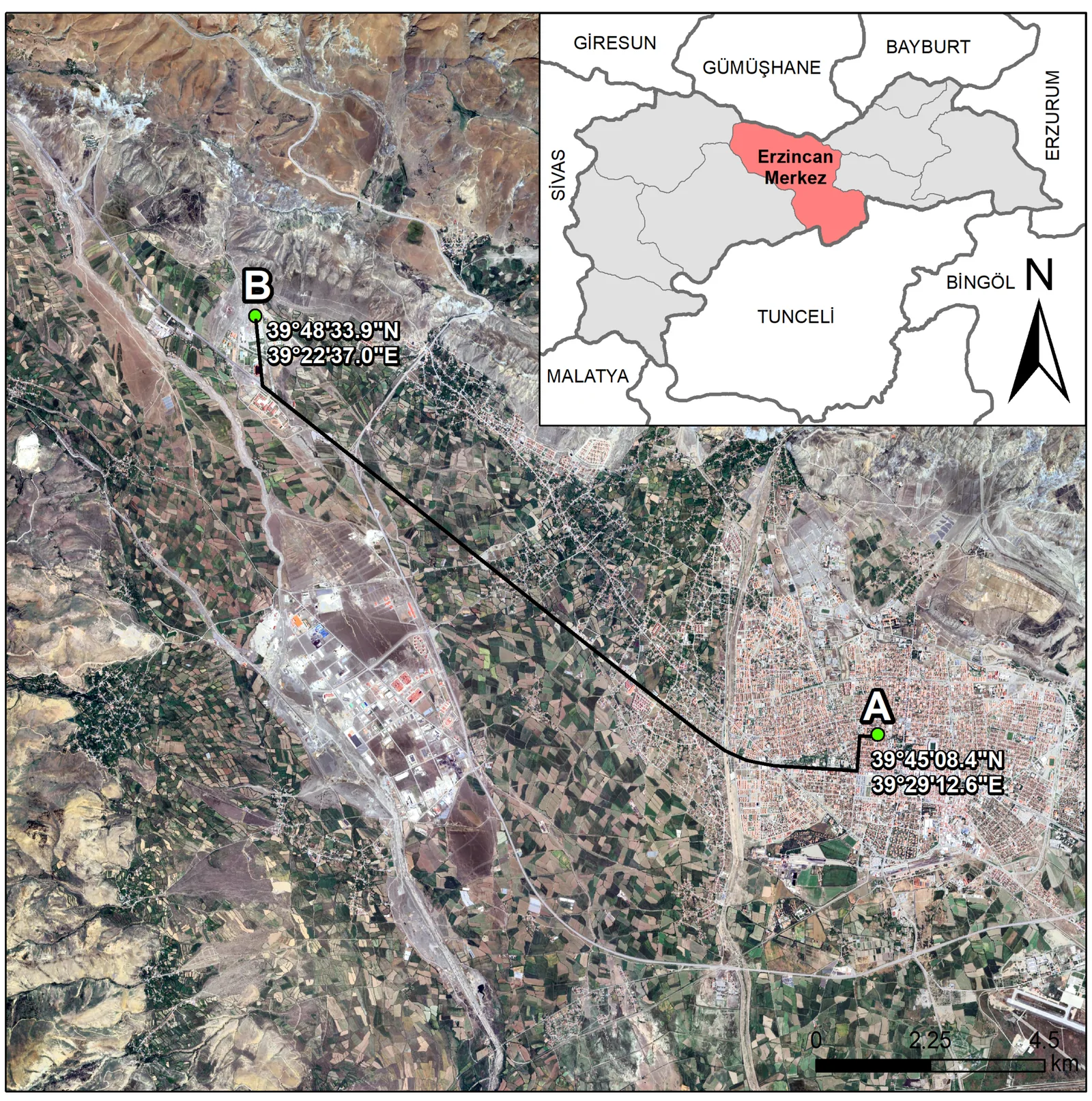
Location map of the meteorological station (A) and the sampling station (B).

The volumetric method was used to determine the airborne pollen in Erzincan. A Lanzoni VPPS 2000 pollen sampler (Bologna, Italy) was installed on the roof of the Central Library of Erzincan Binali Yıldırım University (Figure 8). The Melinex tapes used for pollen sampling were replaced weekly during 2022 and 2023. In the laboratory, the tapes were cut into seven equal pieces of 48 mm, and 24 h preparations were prepared. Pollen grains were counted using the counting method recommended by the Spanish Aerobiology Network (REA). To ensure timely analysis while maintaining representative sampling, four continuous horizontal transects were examined across each daily slide under Leica light microscope (Wetzlar, Germany) at 400× magnification. All identified pollen grains encountered along the transects were recorded, and hourly variations were determined using the standard REA ruler, which divides the daily sampling area into twenty-four 2 mm intervals corresponding to hourly deposition. The pollen concentration per cubic meter of air was then calculated according to the methodologies established by the Spanish Aerobiology Network (REA) [86]. The mean pollen concentration for each taxon was calculated as the arithmetic average of the annual concentrations recorded in 2022 and 2023. Taxon percentages were subsequently calculated using these mean values.

The annual pollen integral (APIn) was determined for each year. Based on the average data from 2022 and 2023, a pollen calendar was prepared to show the periods when pollen was first detected, reached maximum concentration, and ended in the Erzincan atmosphere. The pollen calendar was constructed following the Spieksma model: daily pollen concentrations were summed over 10-day periods, and the averages for the studied years were calculated. These averages were classified into a series of categories of 10-day mean pollen concentrations and represented in the calendar by column heights (Supplementary Materials Figure S3) [87].

Pollen taxa contributing more than 3% of the mean annual pollen integral (APIn) calculated for the two study years were defined as the dominant airborne pollen taxa in Erzincan. For these dominant taxa, the main pollen season (MPS) was calculated using the 95% method proposed by Andersen [73]. According to this method, the MPS was defined as the period between the day when 2.5% of the APIn was reached and the day when 97.5% of the annual pollen concentration was reached, with the start date defined as January 1 [73]. In addition to the Andersen 2.5–97.5% method, the 3d5 concentration-threshold approach was applied to provide a more clearly delimited estimate of the MPS, particularly for taxa with prolonged MPS durations. According to this approach, the MPS started on the first day of a sequence of three consecutive days with daily pollen concentrations of ≥5 pollen/m^3^ and ended on the last day of such a sequence. The MPS duration was calculated as the number of days between the identified start and end dates, inclusive [74].

Daily meteorological data for Erzincan Province, including mean air temperature (°C), mean relative humidity (%), mean wind speed (m s^−1^), and total daily precipitation (mm), were obtained from the Turkish State Meteorological Service (MGM) station, located approximately 9 km from the pollen sampling site. Daily mean meteorological values were calculated by MGM from 24 h measurements, whereas precipitation was provided as the total daily amount. The localities of the pollen sampler installed at the Central Library of Erzincan Binali Yıldırım University and the meteorological station from which the data were collected are shown in Figure 8. MGM station located approximately 9 km from the pollen sampling site at Erzincan Binali Yıldırım University. Both sites are located within the Erzincan Plain and have similar elevations and regional environmental conditions. The station was therefore considered the most appropriate available source for characterizing the regional meteorological conditions at the sampling site. The meteorological variables were recorded at a daily temporal resolution and paired with the corresponding daily airborne pollen concentrations for the statistical analyses.

### 4.3. Statistical Modeling and Analysis

The present study examined the relationships between atmospheric pollen data and meteorological variables. To this end, multifaceted statistical approaches were utilized. Initially, a log(x + 1) transformation was implemented for all pollen species with the objective of mitigating the impact of elevated variation and right-skewness in pollen concentrations. As meteorological variables (temperature, humidity, rainfall, wind speed) were measured on different scales, they were normalized using z-score standardization to ensure their comparability.

Initially, pairwise relationships between pollen types (dominant pollen taxa: Cupressaceae/Taxaceae, Poaceae, Pinaceae, *Artemisia*, Urticaceae, *Quercus*, Amaranthaceae, and *Populus*) and meteorological variables were evaluated using Spearman rank correlation. This step formed the basis for identifying which pairs of variables exhibited significant relationships and the direction of those relationships. Subsequently, multiple linear regression (MLR) and general linear model (GLM) analyses were used to evaluate the relationships between meteorological variables and pollen density. MLR models were applied to assess the independent effects of meteorological parameters on pollen density, with overall model significance evaluated using F-tests and individual regression coefficients assessed using *t*-tests. Within the GLM framework, univariate ANOVA was used to examine differences in meteorological variables among pollen taxa and seasons, while MANOVA and, when appropriate, MANCOVA were applied to evaluate differences in the overall meteorological profile. Homogeneity of variances was assessed using Levene’s test.

Linear mixed models (LMMs) were used to account for the time-series structure and the dependence arising from repeated measurements. LMMs were used to account for temporal dependency and the correlated structure of repeated pollen observations. The repeated-measures structure was defined using days (day index) as the time variable, and temporal autocorrelation between consecutive observations was modeled with an AR(1) covariance structure. A set of candidate models with alternative fixed-effect structures was compared using the Akaike information criterion (AIC), Bayesian information criterion (BIC), marginal R^2^, and AR(1) rho (autoregressive covariance structure of order 1) values. Taxa was also tested as a random-intercept term, but its estimated variance was effectively zero; therefore, Taxa was retained as a fixed effect in the final model.

In the final stage, canonical analyses was performed to examine the shared variance structure between the pollen-species measurements and the meteorological variables. Canonical correlation analysis (CCorA) relationships between linear combinations of the two variable sets were evaluated with canonical correlation coefficients, eigenvalues, Wilks’ Lambda, and associated significance tests. Canonical Correspondence Analysis (CCA) was conducted using R software version 4.1.3 (R Foundation for Statistical Computing, Vienna, Austria) to examine the distribution of pollen taxa along meteorological gradients. The CCA ordination diagram was generated using PAST version 5.3 (Natural History Museum, University of Oslo, Oslo, Norway). Biological seasonal interpretations were derived from the canonical loadings. The findings were reported in terms of statistical significance, effect sizes, and model-fit measures. Across all statistical analyses, *p* < 0.05 was considered to indicate statistical significance. The analyses were performed using SPSS (IBM Corp., Armonk, NY, USA, v30) and Jamovi (The jamovi project, Sydney, Australia, v2.7.28) statistics software.

## 5. Conclusions

In conclusion, the airborne pollen spectrum recorded in Erzincan reflects the combined influence of the city’s geographical location, floristic diversity, urban vegetation, and continental climatic conditions. As the first volumetric aerobiological study conducted in the city, this research provides a fundamental reference dataset for dominant pollen taxa, seasonal distribution patterns, and pollen season timing. In this study, pollen data were evaluated together with seasonal and meteorological variables within a multivariate statistical framework. This approach enables the obtained data to be used as a comparable dataset at the international level for assessing urban pollen dynamics under different climatic and ecological conditions. The findings show that dominant pollen taxa and their seasonal distributions are closely associated with meteorological variables, emphasizing the importance of jointly considering biological, climatic, and urban ecological factors in pollen monitoring studies. This approach may contribute to the development of more reliable pollen calendars, a better interpretation of pollen season dynamics, and the improvement of public health planning and pollen forecasting systems. This study has several limitations that should be acknowledged. Although the findings provide useful preliminary information, multi-year and long-term monitoring is required to obtain more reliable and generalizable results, particularly considering interannual variability in pollen concentrations and meteorological conditions. The present analysis included a limited number of environmental variables. Therefore, future studies should also consider additional factors such as wind direction, wind persistence, atmospheric stability, and other relevant meteorological parameters in order to better explain variability in pollen distribution and transport. With the acquisition of larger and long-term datasets, future research could apply advanced analytical approaches such as time-series analysis, neural network autoregression (NNAR) models, dynamic regression models, and other data-driven techniques. Finally, further research is needed to evaluate the potential contribution of long-range pollen transport events that may affect local pollen concentrations; such processes should be considered in future monitoring and modeling studies. Overall, this study shows that volumetric pollen monitoring, when supported by advanced statistical evaluations, enables airborne pollen dynamics to be assessed in a more explanatory and comparable manner within the context of seasonal, meteorological, and ecological processes. In this respect, the obtained data provide a reference that may contribute both to understanding local pollen exposure and to international monitoring and forecasting studies conducted under different climatic and urban ecological conditions.

## Figures and Tables

**Table 1:** APIn pollen*day/m^3^ and percentage of pollen taxa recorded in Erzincan atmosphere (2022–2023).

Taxa	2022 APIn	2022 (%)	2023 APIn	2023 (%)	Mean APIn	Mean (%)
Cupressaceae/Taxaceae	1816	20.95	3278	24.18	2547	22.92
Pinaceae	672	7.75	1917	14.14	1295	11.65
*Quercus*	459	5.30	577	4.26	518	4.66
*Populus*	493	5.69	356	2.63	425	3.82
*Morus*	367	4.23	195	1.44	281	2.53
*Fraxinus*	212	2.45	206	1.52	209	1.88
*Salix*	233	2.69	176	1.30	205	1.84
Rosaceae	50	0.58	83	0.61	67	0.60
*Ulmus*	45	0.52	62	0.46	54	0.48
*Juglans*	45	0.52	56	0.41	51	0.45
*Alnus*	54	0.62	20	0.15	37	0.33
*Betula*	42	0.48	32	0.24	37	0.33
*Fagus*	53	0.61	14	0.10	34	0.30
*Corylus*	50	0.58	13	0.10	32	0.28
Cistaceae	19	0.22	4	0.03	16	0.27
*Ligustrum*	12	0.14	11	0.08	12	0.10
Ericaceae	2	0.02	17	0.13	10	0.09
*Carpinus*	9	0.10	7	0.05	8	0.07
*Acer*	11	0.13	4	0.03	8	0.07
*Tilia*	8	0.09	3	0.02	6	0.05
Woody	4652	53.67	7031	51.86	5842	52.57
Poaceae	1376	15.88	3421	25.23	2399	21.58
*Artemisia*	1247	14.39	645	4.76	946	8.51
Urticaceae	259	2.99	1056	7.79	658	5.92
Amaranthaceae	502	5.79	394	2.91	448	4.03
*Rumex*	152	1.75	323	2.38	238	2.14
Boraginaceae	81	0.93	149	1.10	115	1.03
*Plantago*	58	0.67	133	0.98	96	0.86
*Xanthium*	68	0.78	81	0.60	75	0.67
Asteraceae	58	0.67	59	0.44	59	0.53
Fabaceae	63	0.73	52	0.38	58	0.52
Apiaceae	31	0.36	41	0.30	36	0.32
Caryophyllaceae	35	0.40	35	0.26	35	0.31
Lamiaceae	14	0.16	45	0.33	30	0.27
Cyperaceae	11	0.13	41	0.30	26	0.23
*Taraxacum*	13	0.15	34	0.25	24	0.21
*Euphorbia*	20	0.23	8	0.06	14	0.13
Herbaceous	3988	46.01	6517	48.07	5253	47.27
Unidentified	27	0.31	10	0.07	19	0.17
Total	8667	100.00	13,558	100.00	11,113	100.00

**Table 2:** MPS of dominant pollen taxa.

Taxa	Year	2.5–97.5% *			3d5 *			Peak Date (d/m)	Max. Pollen Number (pollen/m^3^)
		Start Date (d/m)	End Date (d/m)	Season Length (d)	Start Date (d/m)	End Date (d/m)	Season Length (d)		
Cupressaceae/ Taxaceae	2022	28-Mar	6-Jul	100	28-Mar	5-Jul	99	10-May	214
	2023	4-Mar	4-Jul	122	10-Mar	30-Jun	112	6-May	335
Pinaceae	2022	2-Feb	21-Oct	261	18-May	17-Jul	60	25-May	60
	2023	23-Mar	13-Sep	174	2-May	31-Jul	90	7-Jun	317
*Quercus*	2022	15-Mar	5-Jun	119	20-Apr	26-May	36	26-Apr	35
	2023	24-Apr	6-Jun	43	26-Apr	1-Jun	36	19-May	91
*Populus*	2022	9-Apr	30-Apr	21	11-Apr	24-Apr	13	16-Apr	109
	2023	23-Mar	3-May	41	5-Apr	14-Apr	9	8-Apr	56
Poaceae	2022	23-Apr	3-Nov	225	30-May	11-Oct	134	5-Jul	104
	2023	30-Apr	13-Sep	135	30-Apr	5-Jul	124	4-Jul	110
*Artemisia*	2022	5-Sep	27-Oct	52	3-Oct	20-Oct	17	10-Oct	168
	2023	31-Aug	9-Nov	70	5-Oct	16-Oct	11	10-Oct	102
Urticaceae	2022	11-Apr	25-Sep	167	4-Jul	7-Aug	34	4-Jul	34
	2023	17-May	17-Aug	92	19-May	5-Aug	78	20-May	92
Amaranthaceae	2022	30-Mar	8-Nov	223	8-Aug	4-Oct	57	27-Sep	21
	2023	23-Mar	6-Nov	228	30-Jul	8-Sep	40	27-Aug	17

* 2.5–97.5%, Andersen cumulative percentage method; 3d5, concentration-threshold method.

**Table 3:** Spearman correlations: daily pollen and mean daily meteorological variables (2022–2023).

Met. Parameters	Temperature (°C)		Relative Humidity (%)		Rainfall (mm)		Wind Speed (m/s)	
Taxa	2022	2023	2022	2023	2022	2023	2022	2023
Cupressaceae/Taxaceae	0.188 ***	0.268 ***	−0.275 ***	−0.084	0.003	0.213 ***	0.343 ***	0.283 ***
Poaceae	0.680 ***	0.739 ***	−0.551 ***	−0.479 ***	−0.097	−0.076	0.237 ***	0.394 ***
Pinaceae	0.470 ***	0.429 ***	−0.387 ***	−0.259 ***	−0.025	−0.031	0.277 ***	0.268 ***
*Artemisia*	0.257 ***	0.212 **	−0.142 ***	−0.141 **	−0.111 *	−0.115 *	−0.134 ***	−0.086
Urticaceae	0.441 ***	0.630 ***	−0.429 ***	−0.427 ***	−0.164 ***	−0.087	0.184 ***	0.375 ***
*Quercus*	0.077	0.094	−0.203 ***	0.012	0.040	0.181 **	0.222 ***	0.145 **
Amaranthaceae	0.486 ***	0.601 ***	−0.431 ***	−0.510 ***	−0.203 ***	−0.228 ***	0.025	0.174 ***
*Populus*	0.012	−0.115 *	−0.139 **	0.166 **	0.020	0.214 ***	0.175 ***	0.060

*: *p* < 0.05, **: *p* < 0.01, ***: *p* < 0.001. All tests are two-tailed.

**Table 4:** Fit assessment and selection of candidate linear mixed models.

Model	Predictors	−2LL	AIC	AICC	BIC	R^2^_m_	AR(1) rho
M1	Met	9265.91	9279.91	9279.93	9326.61	0.033	0.83
M2	Year + Met	9262.60	9278.60	9278.62	9331.98	0.038	0.82
M3	Year + Season + Met	9228.44	9250.44	9250.48	9323.84	0.087	0.81
M4	Year + Season + Taxa + Met	9156.06	9192.06	9192.17	9312.16	0.189	0.79
M5	Year + Season + Taxa + Aprp + Met	**8923.98**	**8961.98**	**8962.12**	**9088.76**	**0.358**	**0.73**

Note. Aprp = active pollen release period; −2LL = −2 log likelihood; AIC = Akaike information criterion; AICC = corrected Akaike information criterion; BIC = Bayesian information criterion; R^2^m = marginal R^2^, indicating the proportion of variance explained by fixed effects; AR(1) rho = autocorrelation between successive repeated observations. Met = z-score meteorological variables, including temperature, humidity, rainfall and wind speed. Bold values indicate the best-fitting model.

**Table 5:** Fixed-effect estimates of the linear mixed model.

Parameter	*β*	Std. Error	df	t	*p*	95% CI	
						Low	Upp
Intercept	1.030	0.101	668.4	10.198	<0.001	0.831	1.223
Year	−0.124	0.023	5823	−5.365	<0.001	−0.170	−0.079
Temperature	0.348	0.023	5823	14.957	<0.001	0.302	0.393
Relative humidity	0.012	0.026	5823	0.475	0.635	−0.038	0.062
Rainfall	0.031	0.015	5823	2.038	0.042	0.001	0.060
Wind speed	0.023	0.017	5823	1.345	0.179	−0.011	0.057
Active pollen release period	−0.947	0.056	962.2	−16.877	0.001	−1.057	−0.837
Spring	0.511	0.079	857.9	6.458	<0.001	0.356	0.667
Summer	0.600	0.085	684.4	7.077	<0.001	0.433	0.766
Autumn	0.234	0.079	864.7	2.946	0.003	0.078	0.390
2022 * temp.	−0.220	0.034	5823	−6.514	<0.001	−0.287	−0.154
2022 * humidity	−0.164	0.037	5823	−4.387	<0.001	−0.237	−0.091
2022 * rainfall	0.059	0.031	5823	1.925	0.054	−0.001	0.119
2022 * wind speed	−0.010	0.025	5823	−0.402	0.688	−0.058	0.039
*Artemisia* * autumn	0.477	0.233	1081.3	2.044	0.041	0.019	0.934
Cupressaceae * spring	1.484	0.236	967.9	6.293	<0.001	1.021	1.947
Pinaceae * summer	0.505	0.267	1045.1	1.888	0.050	−0.020	1.030
Poaceae * summer	0.713	0.274	1080.9	2.602	0.009	0.175	1.251
*Quercus* * summer	−0.549	0.250	933.8	−2.196	0.028	−1.039	−0.058

**Table 6:** Summary statistics for the four axes of CCA conducted on eight pollen taxa using four met. variables.

Parameters	Axis				Total Inertia
	1	2	3	4	
Eigenvalues	0.154	0.039	0.012	0.001	
Species–environment correlations	0.588	0.284	0.247	0.059	
Percentage variance of species data explained by each axis (%)	8.55	2.21	0.70	0.05	
Cumulative percentage variance of species data (%)	8.55	10.77	11.46	11.51	
Percentage variance of species–environment relation explained by each axis (%)	74.26	19.23	6.05	0.46	
Cumulative percentage variance of species–environment relation (%)	74.26	93.49	99.54	100.00	
Permutation *p*-value of axes	0.001	0.001	0.003	0.875	
F-value of axes	60.104	15.568	4.897	0.371	
Sum of all canonical eigenvalues/constrained inertia					0.207
Unconstrained inertia					1.592
Total constrained variation explained by climatic variables (%)					11.51
Unconstrained variation (%)					88.49
Interset correlations of climatic variables with CCA axes					VIF
Temperature (*T*)	0.565	−0.070	−0.029	0.003	1.9418
Humidity (*H*)	−0.351	0.067	0.1651	0.022	2.5042
Rainfall (*R*)	−0.243	−0.113	0.017	0.048	1.3730
Wind speed (*Ws*)	0.025	−0.242	0.055	−0.028	1.0798

CCA, Canonical Correspondence Analysis; VIF, variance inflation factor.

## Data Availability

The original contributions presented in this study are included in the article. Further inquiries can be directed at the corresponding author.

## Supplementary Materials

The following supporting information can be downloaded at https://www.mdpi.com/article/10.3390/plants15162476/s1. Supplementary Materials Table S1. Monthly distribution of airborne pollen in Erzincan, 2022 (%). Supplementary Materials Table S2. Monthly distribution of airborne pollen in Erzincan, 2023 (%). Supplementary Materials S3. Multiple linear regression (MLR) and general linear model (GLM) analysis. Supplementary Materials Figure S1. Daily pollen concentration of most dominant woody taxa for the atmosphere of Erzincan: (A) Cupressaceae/Taxaceae, (B) Pinaceae, (C) *Quercus*, (D) *Populus*. Supplementary Materials Figure S2. Daily pollen concentration of most dominant herbaceous taxa for the atmosphere of Erzincan: (A) Poaceae, (B) *Artemisia*, (C) Urticaceae, (D) Amaranthaceae. Supplementary Materials Figure S3. Pollen calendar for Erzincan province (average APIn values of 2022 and 2023).

## Abbreviations

The following abbreviations are used in this manuscript:

APIn	Annual Pollen Integral
AR(1)	Autocorrelation Between Successive Repeated Observations
CORINE	Coordination of Information on the Environment
MPS	Main Pollen Season
REA	Spanish Aerobiology Network
GLM	General Linear Model
LMM	Linear Mixed Model
CCorA	Canonical Correlation
CCA	Canonical Correspondence Analysis
AIC	Akaike Information Criterion
BIC	Bayesian Information Criterion
MANCOVA	Multivariate Analysis of Covariance
ANOVA	Analysis of Variance
ARPR	Active Pollen Release Period
MGM	Turkish State Meteorological Service
