# Supplementary material for: Allergenic Pollen Dynamics in Continental-Climate Urban Ecosystem: Multivariate Statistical Modeling of a 24-Month Observational Study
Source: Plants (Basel). 2026 Aug 15;15(16):2476. doi: 10.3390/plants15162476 (PMC13516413; doi:10.3390/plants15162476)
Supplement: Supplementary file 1 [file plants-15-02476-s001.zip › Supplementary Material Table S1. Monthly distribution of airborne pollen in Erzincan, 2022 (%).pdf]

Table S1. Monthly distribution of airborne pollen in Erzincan, 2022 (%)

**Taxa**	**Jan-22**	**Feb-22**	**Mar-22**	**Apr-22**	**May-22**	**Jun-22**	**Jul-22**	**Aug-22**	**Sep-22**	**Oct-22**	**Nov-22**	**Dec-22**	**Total (%)**
**Cupressaceae/Taxaceae**	0,06	0,06	1,02	6,14	11,41	1,13	1,07	0,02		0,01		0,03	20,95
**Pinaceae**	0,18	0,05	0,07	0,30	2,46	1,87	1,75	0,35	0,42	0,20	0,10	0,01	7,75
Populus			0,01	5,60	0,08								5,69
Quercus			0,03	2,09	2,84	0,33							5,30
Morus			0,08	1,12	3,03								4,23
Salix		0,02	0,09	1,60	0,89	0,08							2,69
Fraxinus			0,45	1,95	0,05								2,45
Alnus		0,10	0,47	0,05									0,62
Fagus	0,02	0,03	0,05	0,50	0,01								0,61
Corylus			0,23	0,30	0,05								0,58
**Rosaceae**	0,01	0,02	0,02	0,16	0,02	0,02	0,25	0,05	0,01				0,58
Juglans				0,14	0,38								0,52
Ulmus			0,23	0,29									0,52
Betula		0,01	0,45	0,01	0,01								0,48
**Cistaceae**				0,18	0,02		0,01						0,22
Ligustrum				0,01	0,03	0,06		0,02	0,01				0,14
Acer				0,12		0,01							0,13
Carpinus				0,06	0,05								0,10
Tilia					0,09								0,09
**Ericaceae**					0,01	0,01							0,02
**Woody**	0,28	0,30	3,21	20,61	21,43	3,52	3,09	0,45	0,44	0,21	0,10	0,05	53,67
**Poaceae**	0,08	0,05	0,12	0,40	1,67	1,30	6,90	3,28	1,25	0,40	0,36	0,07	15,88
Artemisia							0,02	0,30	0,53	13,21	0,27	0,06	14,39
Amaranhtaceae	0,05	0,06	0,05	0,03	0,05	0,03	0,23	2,52	1,78	0,80	0,17	0,03	5,79
**Urticaceae**			0,02	0,25	0,15	0,12	1,23	0,98	0,20	0,02	0,01		2,99
Rumex			0,02	0,33	0,75	0,12	0,31	0,15	0,07				1,75
Boraginaceae	0,02			0,02	0,23	0,05	0,52	0,09					0,93
Xanthium								0,24	0,42	0,05	0,07	0,01	0,78
**Fabaceae**				0,25	0,03	0,01	0,09	0,30	0,03				0,73
**Asteraceae**	0,06		0,02	0,02	0,01	0,06	0,06	0,28	0,13		0,02	0,01	0,67
Plantago			0,02	0,03	0,06	0,03	0,38	0,08	0,06				0,67
Caryophyllaceae				0,07	0,01	0,03	0,03	0,17	0,07	0,01			0,40
**Apiaceae**						0,01		0,25	0,06	0,02	0,01		0,36
Euphorbia								0,13	0,06	0,03	0,01		0,23
**Lamiaceae**				0,01	0,05	0,01		0,08	0,01				0,16
Taraxacum				0,03	0,02	0,01	0,01	0,02	0,03		0,01		0,15
**Cyperaceae**		0,01			0,03		0,07	0,01					0,13
**Herbaceous**	0,23	0,12	0,25	1,48	3,05	1,79	9,87	8,88	4,67	14,55	0,95	0,18	46,01
Unidentified	0,06	0,01	0,01	0,06	0,02	0,12	0,02	0,01					0,31
Total	0,57	0,43	3,47	22,14	24,50	5,42	12,98	9,35	5,11	14,76	1,05	0,23	100,00
