# Supplementary material for: Allergenic Pollen Dynamics in Continental-Climate Urban Ecosystem: Multivariate Statistical Modeling of a 24-Month Observational Study
Source: Plants (Basel). 2026 Aug 15;15(16):2476. doi: 10.3390/plants15162476 (PMC13516413; doi:10.3390/plants15162476)
Supplement: Supplementary file 1 [file plants-15-02476-s001.zip › Supplementary Material Table S2. Monthly distribution of airborne pollen in Erzincan, 2023 (%).pdf]

Table S2. Monthly distribution of airborne pollen in Erzincan, 2023 (%)

Taxa	Jan-23	Feb-23	Mar-23	Apr-23	May-23	Jun-23	Jul-23	Aug-23	Sep-23	Oct-23	Nov-23	Dec-23	Total (%)
**Cupressaceae/Taxaceae**	0,02	0,07	3,54	4,54	11,40	3,34	1,01	0,13	0,10	0,01	0,02	0,01	24,18
**Pinaceae**	0,20	0,10	0,19	0,13	1,24	10,15	1,34	0,33	0,19	0,17	0,07	0,02	14,14
Quercus				0,49	3,40	0,36							4,26
Populus			0,15	2,46	0,01								2,63
Fraxinus		0,04	1,36	0,10		0,01							1,52
Morus		0,02	0,12	0,30	1,00								1,44
Salix			0,12	0,29	0,87	0,02							1,30
**Rosaceae**			0,01	0,03	0,01	0,22	0,24	0,08	0,01				0,61
Ulmus			0,44	0,02									0,46
Juglans			0,01	0,06	0,35								0,41
Betula			0,15	0,06	0,02								0,24
Alnus		0,01	0,12	0,01									0,15
**Ericaceae**						0,07	0,06						0,13
Fagus			0,04		0,07								0,10
Corylus	0,01	0,02	0,05			0,01	0,01						0,10
Ligustrum					0,02	0,05	0,01						0,08
Carpinus			0,04		0,01								0,05
Acer					0,01		0,01	0,01					0,03
**Cistaceae**						0,03							0,03
Tilia							0,01		0,01				0,02
**Woody**	0,23	0,27	6,34	8,50	18,41	14,26	2,70	0,55	0,31	0,18	0,10	0,04	51,86
**Poaceae**	0,08	0,04	0,15	0,38	3,18	9,52	8,70	2,29	0,46	0,21	0,16	0,05	25,23
**Urticaceae**					2,27	1,77	2,91	0,79	0,04	0,01	0,01		7,79
Artemisia							0,01	0,16	0,15	4,26	0,15	0,02	4,76
**Amaranhtaceae**	0,14	0,01	0,02	0,02	0,02	0,07	0,35	1,36	0,70	0,13	0,07	0,02	2,91
Rumex			0,04	0,17	0,82	0,83	0,35	0,15	0,01				2,38
**Boraginaceae**				0,01	0,27	0,27	0,52	0,04					1,10
Plantago			0,08	0,03		0,24	0,51	0,12		0,01			0,98
Xanthium								0,30	0,24	0,01	0,04	0,01	0,60
Asteraceae	0,01		0,01	0,01	0,04	0,06	0,13	0,12	0,04	0,01			0,44
**Fabaceae**	0,01				0,01	0,15	0,14	0,05	0,01		0,01		0,38
**Lamiaceae**					0,02	0,10	0,18	0,01	0,01				0,33
**Apiaceae**				0,01	0,07	0,06	0,11	0,04	0,01				0,30
**Cyperaceae**			0,01	0,01	0,08	0,09	0,10	0,02					0,30
**Caryophyllaceae**					0,04	0,03	0,11	0,06	0,02				0,26
Taraxacum				0,01	0,10	0,07	0,04	0,01	0,01	0,01			0,25
Euphorbia							0,04	0,01	0,01				0,06
**Herbaceous**	0,24	0,04	0,32	0,66	6,93	13,26	14,20	5,52	1,71	4,65	0,44	0,10	48,07
Unidentified	0	0,01	0,01	0	0,01	0,01	0,02	0	0	0	0	0	0,07
Total	0,49	0,32	6,68	9,15	25,4	27,5	16,9	6,03	2,02	4,82	0,54	0,14	100,00
