# Supplementary material for: Allergenic Pollen Dynamics in Continental-Climate Urban Ecosystem: Multivariate Statistical Modeling of a 24-Month Observational Study
Source: Plants (Basel). 2026 Aug 15;15(16):2476. doi: 10.3390/plants15162476 (PMC13516413; doi:10.3390/plants15162476)
Supplement: Supplementary file 1 [file plants-15-02476-s001.zip › Supplemetary Metarial S3.pdf]

2.2.2. Multiple linear Regression (MLR) and General Linear Model (GLM) Analysis

The multiple linear regression model established to explain the pollen concentration is statistically significant (F (4, 5835)=147.99; p<0.001) and the model variance is R^2=0.092 (adj. R^2:0.091). Variance inflation factors (VIF) for the model predictors were as follows: temperature = 2.182; humidity = 2.645; rainfall = 1.271; and wind speed (Ws) = 1.156. The findings indicate that temperature (β=0.26 p<0.001) and rainfall (β = 0.059, p<0.001) have significant positive effects on pollen density, whereas humidity (β=−0.055 p=0.007) has a significant negative effect. The effect of wind speed was not statistically significant (*β*=−0.019 *p*=0.155). The findings indicate that increases in temperature and rainfall can raise pollen density, while high humidity can reduce it to a limited extent. When all species were evaluated together, temperature (*p*<0.001), humidity (p=0.007), and rainfall (*p*< 0.001) had statistically significant effects on pollen density. The largest effect size was observed for temperature, which accounted for 3.3% of the variance (η^2_p=0.033). Rainfall had a smaller but still significant effect (η^2_p=0.003), and the effect of humidity was very small (η^2_p=0.001). Wind speed did not have a significant influence on the outcomes. Species-based evaluation revealed that temperature significantly affected *Poaceae, Urticaceae, Pinaceae, and Amaranthaceae*; rainfall affected *Cupressaceae/Taxaceae, Quercus*, and *Populus*; wind speed affected *Artemisia, Amaranthaceae*, and *Cupressaceae/Taxaceae*; and humidity affected A*maranthaceae, Populus*, and *Quercus*, indicating that meteorological variables have species-specific effects on pollen production (Table 1.).

Table 1. Effects of meteorological variables by species of taxa.

Taxa	P-value				(η^2_p)
	**Temp.**	**Humidity**	**Rainfall**	**Wind Sp.**	
Cupressaceae/Taxaceae	0.052	0.134	<0.001	0.005	Temp. (0.021)
Poaceae	<0.001	0.688	0.455	0.117	Temp. (0.245)
Pinacea	<0.001	0.435	0.220	0.167	Temp. (0.085)
Artemisia	<0.001	0.550	0.183	<0.001	Temp. (0.022)
Urticeae	<0.001	0.331	0.407	0.076	Temp. (0.088)
Quercus	0.830	0.013	0.003	0.178	Rainfall (0.012)
Amaranthaceae	<0.001	<0.001	0.739	<0.001	Temp. (0.106)
Populus	0.024	0.024	<0.001	0.290	Rainfall (0.027)

In the general linear model (GLM), pollen concentration was modeled with species and meteorological variables as fixed effects, with species included to account for interspecific differences. The effects of meteorological variables on pollen density, species differences, and species × meteorological interactions were assessed. Statistical modeling indicates that pollen density is significantly influenced by both meteorological variables and interspecies differences (F (39, 5839) =10.85, R^2 = 0.068 (adj.R^2:0.062), η^2_p:0.068, p<0.001). Statistical findings show that pollen–climate relationships cannot be explained solely by general meteorological trends, and that species respond differently to these variables. Temperature is the predictor of overall pollen density (*p*<0.001), but the magnitude and direction of its effect vary considerably among species. Species in the Poaceae show a high sensitivity to rising temperatures, suggesting that sudden increases in pollen production during warmer periods may raise the risk of allergenicity. However, in Populus, increasing temperatures reduce pollen production, suggesting that this genus maximizes pollen output under cooler conditions or that higher temperatures constrain its phenological cycle. Although rainfall had a weak but

significant positive effect (*p*=0.034), it suggests that humid conditions tend to increase pollen production in Cupressaceae/Taxaceae species and that allergen load may rise during rainy periods (*p*=0.037). Humidity and wind were not significant in the overall model, yet biologically meaningful trends were observed for some species, indicating that microclimatic conditions and local adaptations also influence pollen production. These findings indicate that pollen density is shaped not only by meteorological conditions but also by the species' specific phenological and ecological characteristics (Table 2).

Table 2. GLM fixed effects and significant species × meteorology interactions.

Effect type	Variable/ interaction	F	p	η^2_p	Comments
**Fixed effect**	Temperature	38.205	<0.001	0,007	Temperature most drives higher pollen concentration; pollen increases as temperature rises.
	Rainfall	4.479	0.034	0.001	Significant positive effect.
	Relative humidity	1.657	0.198	<0.001	Not significant.
	Wind Speed	0.103	0.748		
	Taxa	2.067	0.044	0,002	Overall differences in pollen among species
**Taxa x Meteorolog.**	*Poaceae* x temp.	+0.499	<0.001	Rising temperature increases *Poaceae* pollen.	
	Populus x temp.	–0.236	0.023	Temperature increase reduces *Populus* pollen.	
	Cupressaceae x rainfall	+0.504	0.037	Increased rainfall increases Cupressaceae pollen production.	
**Taxa main effect**	Cupressaceae	+11.658	0.020	Significantly higher pollen production compared with *Urticeae*.	
